# MoImd4 mediates crosstalk between MoPdeH‐cAMP signalling and purine metabolism to govern growth and pathogenicity in *Magnaporthe oryzae*

**DOI:** 10.1111/mpp.12770

**Published:** 2019-01-11

**Authors:** Lina Yang, Yanyan Ru, Xingjia Cai, Ziyi Yin, Xinyu Liu, Yuhan Xiao, Haifeng Zhang, Xiaobo Zheng, Ping Wang, Zhengguang Zhang

**Affiliations:** ^1^ Department of Plant Pathology, College of Plant Protection Nanjing Agricultural University, and Key Laboratory of Integrated Management of Crop Diseases and Pests, Ministry of Education Nanjing 210095 China; ^2^ Departments of Pediatrics, and Microbiology, Immunology, and Parasitology Louisiana State University Health Sciences Center New Orleans LA 70112 USA

**Keywords:** GTP biosynthesis, inosine‐5′‐monophosphate dehydrogenase, *Magnaporthe oryzae*, pathogenicity, phosphodiesterase

## Abstract

The high‐affinity cyclic adenosine monophosphate (cAMP) phosphodiesterase MoPdeH is important not only for cAMP signalling and pathogenicity, but also for cell wall integrity (CWI) maintenance in the rice blast fungus *Magnaporthe oryzae*. To explore the underlying mechanism, we identified MoImd4 as an inosine‐5′‐monophosphate dehydrogenase (IMPDH) homologue that interacts with MoPdeH. Targeted deletion of *MoIMD4* resulted in reduced *de novo* purine biosynthesis and growth, as well as attenuated pathogenicity, which were suppressed by exogenous xanthosine monophosphate (XMP). Treatment with mycophenolic acid (MPA), which specifically inhibits MoImd4 activity, resulted in reduced growth and virulence attenuation. Intriguingly, further analysis showed that MoImd4 promotes the phosphodiesterase activity of MoPdeH, thereby decreasing intracellular cAMP levels, and MoPdeH also promotes the IMPDH activity of MoImd4. Our studies revealed the presence of a novel crosstalk between cAMP regulation and purine biosynthesis in *M. oryzae*, and indicated that such a link is also important in the pathogenesis of *M. oryzae.*

## Introduction

In the rice blast fungus *Magnaporthe oryzae*, the cyclic adenosine monophosphate (cAMP) signalling pathway plays an important role in vegetative growth, asexual/sexual development, cell wall integrity (CWI), appressorium formation and virulence (Lee and Dean, 1993; Liu *et al.*, 2016b; Ramanujam and Naqvi, 2010; Yang *et al.*, 2017; Yin *et al.*, 2016; Zhang *et al*., 2011a, 2011b). In *M. oryzae*, intracellular cAMP levels are governed by the dynamic balance between adenylyl cyclase MoMac1, which synthesizes cAMP, and high‐affinity phosphodiesterase MoPdeH and low‐affinity MoPdeL, which hydrolyse cAMP (Ramanujam and Naqvi, 2010; Woobong Choi, 1997; Yang *et al.*, 2017; Zhang *et al.*, 2011aa). Previously, we have found that MoPdeH plays a role in hyphal autolysis, surface signal recognition, conidium morphology, CWI and pathogenicity, whereas MoPdeL appears to affect conidial morphology (Zhang *et al.*, 2011aa). We also found that the HD and EAL domains of MoPdeH are critical for its phosphodiesterase activity (Yang *et al.*, 2017). Interestingly, we found that the protein phosphatase MoYvh1 functions upstream of MoPdeH to regulate CWI and pathogenicity (Liu *et al.*, 2016b); however, the underlying mechanism of this regulation is unclear.

To explore how MoPdeH might affect CWI independent of cAMP signalling, we screened for proteins interacting with MoPdeH, and identified MoImd4 as an inosine‐5′‐monophosphate dehydrogenase (IMPDH) homologue from *M. oryzae*. In eukaryotic cells, IMPDH is involved in *de novo* purine biosynthesis, a highly conserved biological process that provides ATP and GTP energy sources for the cell (Elion, 1989). IMPDH catalyses and hydrolyses inosine monophosphate (IMP) as xanthosine monophosphate (XMP), a rate‐limiting and first committed step in the *de novo* biosynthesis of GTP (Buey *et al.*, 2015b). IMPDH also provides the obligatory precursors for DNA and RNA biosynthesis and cell proliferation, which may be linked to malignant cell transformation or tumour progression (Buey *et al.*, 2015b; Collart and Huberman, 1988; Jackson *et al.*, 1975; Shimura *et al.*, 1983). In the budding yeast *Saccharomyces cerevisiae*, IMPDH is encoded by a family of four genes, named *ScIMD1* to *ScIMD4*, and loss of the *ScIMD* gene family results in cells auxotrophic for guanine. Despite encoding proteins with high amino acid sequence identity, their functions are distinct: *ScIMD1* is a pseudogene, ∆*Scimd2 *has intrinsic drug resistance, and ∆*Scimd3 *and ∆*Scimd4 *confer drug resistance lacking *ScIMD2* (Hyle *et al.*, 2003). In *Cryptococcus neoformans*, CnImd1 is important for growth, synthesis of the cryptococcal polysaccharide capsule and melanin, and virulence in mouse and nematode models (Morrow *et al.*, 2012). In *Ashbya gossypii*, overexpression of the *IMPDH* gene increases metabolic flux through the guanine pathway, which ultimately enhances riboflavin production (Buey *et al.*, 2015a).

IMPDH contains two conserved tandem cystathionine β‐synthase (CBS) subdomains (Bateman, 1997) which are also present in a variety of proteins, including voltage‐gated chloride channels and the AMP‐activated protein kinase (Baykov *et al.*, 2011; Ignoul and Eggermont, 2005). The CBS domain mutation is related to many hereditary diseases of humans, such as homocystinuria, Wolff–Parkinson–White syndrome and congenital myotonia (McGrew and Hedstrom, 2012; Scott *et al.*, 2004). In *Escherichia coli*, CBS domains are essential for the global regulation of purine utilization, ATP/GTP ratio and function of the enzymatic activity of IMPDH (Pimkin and Markham, 2008). However, not all IMPDH CBS domains are accountable for specific defects, such as in *Pseudomonas aeruginosa* and *C. neoformans* (Morrow *et al.*, 2012; Rao *et al.*, 2013)*.* Hence, it seems that the physiological functions of CBS domains vary considerably between different organisms.

Previous studies have found that MoPdeH plays a multifaceted role in *M. oryzae* (Ramanujam and Naqvi, 2010; Woobong Choi, 1997; Yang *et al.*, 2017; Zhang *et al.*, 2011aa). Here, we continue to investigate the mechanism linking MoPdeH to fungal pathogenesis. We identified a MoPdeH‐interacting protein, MoImd4, through a yeast two‐hybrid screen, and found that MoImd4 is involved in the *de novo* purine metabolic pathway. MoImd4 and MoPdeH regulate their enzymatic activities mutually to promote the development and pathogenicity of *M. oryzae.* The finding of MoImd4 in association with MoPdeH provides a new link between cAMP signalling and the purine biosynthesis pathway. It also reveals that MoImd4 has functions beyond guanine nucleotide biosynthesis.

## Results

### Identification of MoImd4 as an MoPdeH‐interacting protein

**Figure 1:**
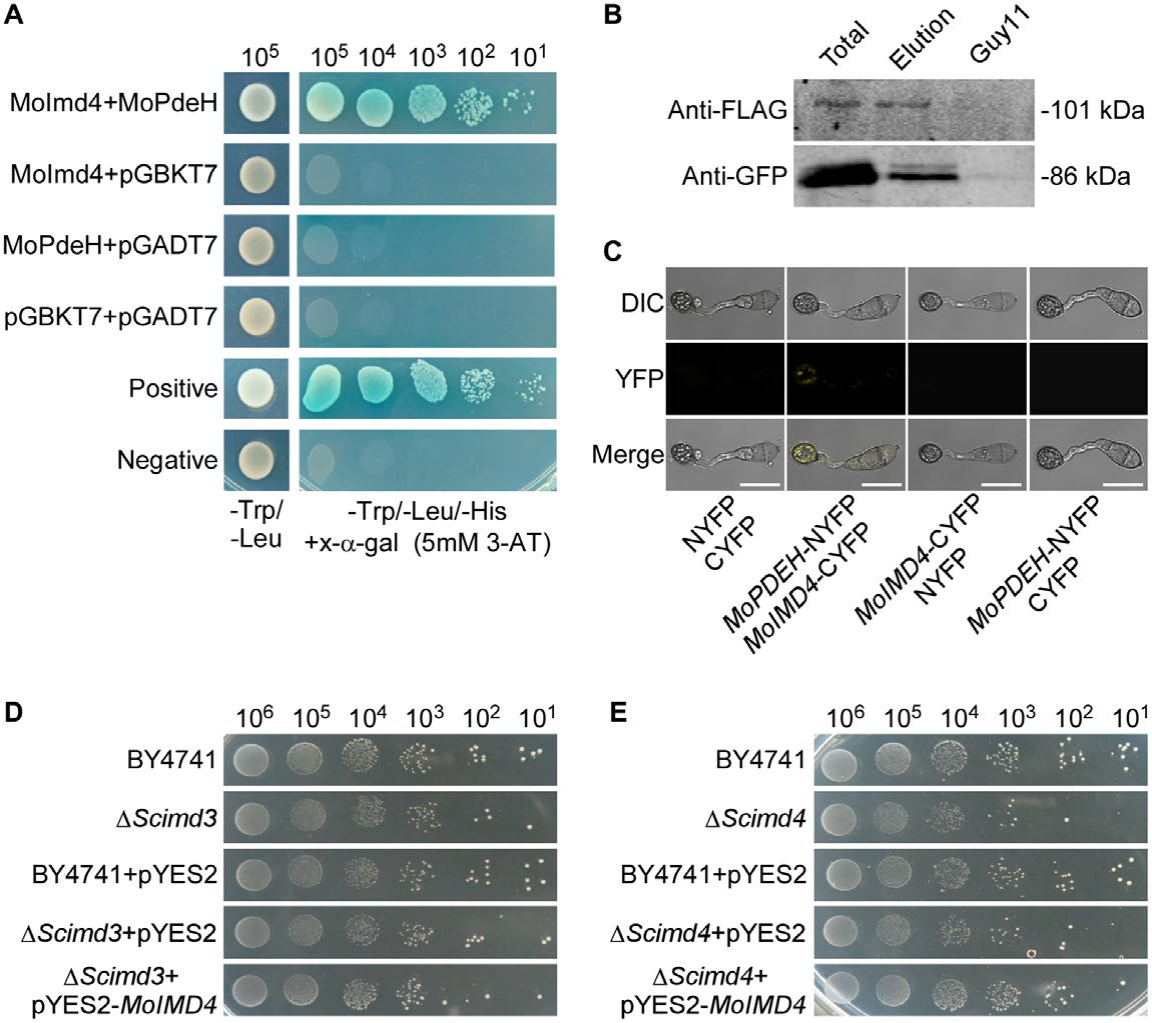
MoPdeH physically interacts with MoImd4. (A) Yeast two‐hybrid assay of the interaction between MoImd4 and MoPdeH. *MoIMD4* was inserted into vector pGADT7 and *MoPDEH* was inserted into vector pGBKT7. Both vectors were co‐transferred into yeast AH109 cells and incubated on SD‐Leu‐Trp for 3 days prior to selection on SD‐Leu‐Trp‐His medium with 1 mM X‐α‐gal and 5 mM 3‐amino‐1,2,4‐triazole (3‐AT) for a further 3 days. (B) Co‐immunoprecipitation (Co‐IP) assay for the interaction between MoImd4 and MoPdeH. Plasmids of *MoPDEH*‐Flag and *MoIMD4*‐GFP were co‐expressed in wild‐type Guy11, and proteins were detected using anti‐Flag and anti‐GFP antibodies. Lysed hyphal proteins were allowed to bind to Flag beads at 4 °C for 4 h and analysed by immunoblot (IB) with appropriate antibodies. GFP, green fluorescent protein. (C) Bimolecular fluorescence complementation (BiFC) assay for the interaction between MoImd4 and MoPdeH. Transformants expressing *MoIMD4*‐CYFP and *MoPDEH*‐NYFP were analysed by differential interference contrast (DIC) and epifluorescence microscopy following incubation on hydrophobic slides at 8 h post‐inoculation (hpi). YFP, yellow fluorescent protein. Bar, 10 μm. (D, E) MoImd4 partially rescues the growth defect of ∆*Scimd4*, but not of ∆*Scimd3*. Serial dilutions of BY4741, ∆*Scimd3*, ∆*Scimd4*, pYES2 and pYES2‐*MoIMD4* transformants were grown on SD‐Leu‐Met‐Ura‐His (galactose) plates at 30 °C for 6 days.

MoPdeH is a high‐affinity phosphodiesterase that hydrolyses intracellular cAMP required for the vegetative growth, functional appressorium development and virulence of *M. oryzae* (Ramanujam and Naqvi, 2010; Yang *et al.*, 2017; Zhang *et al.*, 2011aa). To further explore the molecular regulatory mechanism of MoPdeH, we screened a yeast two‐hybrid cDNA library constructed with an RNA pool from various stages, including conidia and infectious hyphae (0, 2, 4, 8, 12 and 24 h). Nineteen putative MoPdeH‐interacting proteins were identified. Of these, the fragments of MGG_03699 showed the highest frequency (12 times). Thus, we chose MGG_03699 for further characterization. MGG_03699 shares high amino acid sequence homology with inosine monophosphate dehydrogenases (Imds) from various species (Figs 1A, S2 and Table S1, see Supporting Information). To test whether this Imd homologue encodes conserved functions, we expressed the protein with the yeast expression vector pYES2 in ∆*Scimd3* and ∆*Scimd4* strains, and found that the *M. oryzae* protein could partially rescue the growth defect of ∆*Scimd4* (Fig. 1D,E). We named this single IMPDH orthologue of *M. oryzae* as MoImd4. We also validated the interaction between MoImd4 and MoPdeH by co‐immunoprecipitation (Co‐IP) and bimolecular fluorescence complementation (BiFC) assays (Fig. 1B,C).

### MoImd4 is involved in vegetative growth, conidiation and virulence

To examine MoImd4 functions, we generated the ∆*Moimd4* mutant using the standard one‐step gene replacement strategy and also complemented the mutant with the wild‐type *MoIMD4 *gene (Fig. S1, see Supporting Information). Phenotypic analysis showed that MoImd4 is required for mycelial growth, conidial formation and pathogenicity. In comparison with Guy11, the ∆*Moimd4 *mutant showed significantly reduced growth on complete medium (CM), minimal medium (MM), straw decoction and corn agar media (SDC) and oatmeal media (OM) plates and reduced biomass in liquid CM [Fig. S3A,B (see Supporting Information) and Table 1]. Conidiation was also significantly reduced to approximately 0.04‐fold of the wild‐type and the complemented strains following 10 days of growth on SDC medium (Table 1).

**Table 1:** Vegetative growth, biomass and conidiation analysis of the wild‐type, ∆*Moimd4 *mutant and complemented strains.

Strain	Colony diameter (cm)†				Biomass (g)‡	Conidiation (×100/cm^2^)§
	CM	MM	OM	SDC		
Guy11	4.9 ± 0.1	4.0 ± 0.1	4.4 ± 0.1	3.3 ± 0.1	0.0815 ± 0.0028	478.7 ± 36.2
∆*Moimd4*	2.8 ± 0.1*	3.1 ± 0.2*	3.8 ± 0.1*	2.7 ± 0.1*	0.0207 ± 0.0015*	20.2 ± 5.0*
MoImd4	4.8 ± 0.1	3.9 ± 0.1	4.4 ± 0.1	3.3 ± 0.1	0.0803 ± 0.0012	490.4 ± 37.8

±Standard deviation (SD) was calculated from three repeated experiments and asterisks indicate statistically significant differences (Duncan’s new multiple range test, *P < 0.01).

^†^Colony diameter of the indicated strains on complete medium (CM), minimal medium (MM), oatmeal media (OM) and straw decoction and corn agar media (SDC) after 7 days of incubation at 28 °C.

^‡^Dry weight of hyphae at day 2 after incubation in liquid CM with shaking at 160 rpm at 28 °C.

^§^Quantification of the conidial production of the indicated strains from SDC cultures in the dark for 7 days, followed by incubation under constant illumination for 3 days at room temperature.

**Figure 2:**
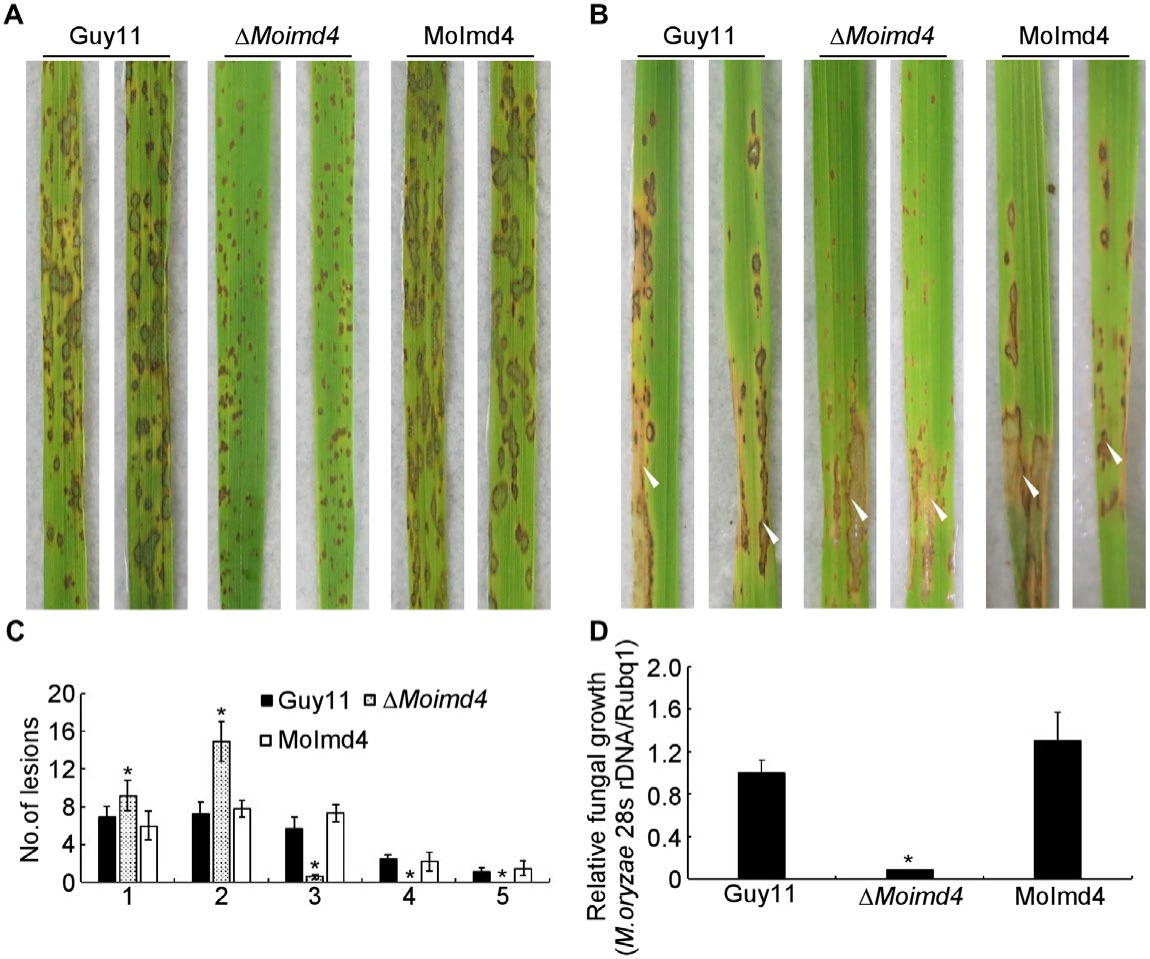
MoImd4 is required for pathogenicity. (A) Conidial suspensions (5 × 10^4^ spores/mL) of Guy11, ∆*Moimd4* and the complemented strains were sprayed onto 11‐day‐old rice seedlings and photographs were taken at 7 days post‐inoculation (dpi). (B) Conidial suspensions (15 × 10^4^ spores/mL) were injected into 18‐day‐old rice sheaths and photographs were taken at 5 dpi. (C) Lesion type statistical analysis (0, no lesion; 1, pinhead‐sized brown specks; 2, 1.5‐mm brown spots; 3, 2–3‐mm grey spots with brown margins; 4, many elliptical grey spots longer than 3 mm; 5, coalesced lesions infecting 50% or more of the leaf area). Lesions were photographed and measured at 7 dpi. Experiments were repeated three times with similar results. Asterisks represent significant differences (Duncan’s new multiple range test, *P* < 0.01). (D) The severity of lesions was analysed by quantification of the *Magnaporthe oryzae* genomic 28S rDNA relative to rice genomic Rubq1 DNA. Experiments were repeated three times with similar results. Error bars represent the standard deviations and asterisk represents significant difference (Duncan’s new multiple range test, *P* < 0.01).

To test the role of MoImd4 in pathogenicity, conidial suspensions of the wild‐type Guy11, ∆*Moimd4* mutant and complemented strains were sprayed or injected into susceptible rice seedlings CO‐39. After 7 days in a chamber at 28 °C with 90% humidity, the ∆*Moimd4* mutant produced smaller and needle‐like lesions compared with the numerous typical lesions produced by the wild‐type and complemented strains (Fig. 2A,B). Meanwhile, a ‘lesion type’ scoring assay according to Liu *et al.* (2016b) showed that the ∆*Moimd4* mutant formed type 1 and 2, very few type 3, and no type 4 or 5 lesions (Fig. 2C). The results of fungal biomass assays in diseased leaves were in accordance with the spraying assays (Fig. 2D). In addition, appressorium formation on the inductive or non‐inductive surface after 24 h was examined and revealed no significant differences between the ∆*Moimd4* mutant and the wild‐type strains (Table 2). Meanwhile, a turgor assay showed that the ∆*Moimd4* mutant was more sensitive than control strains to 1–4 m glycerol (Table 2). Taken together, these results show that MoImd4 is important for the growth, conidiation and pathogenicity of *M. oryzae.*

**Table 2:** Turgor assays and appressorium formation of the wild type, ∆*Moimd4* mutant, and the complement strains.

Strain	Appressorium exhibiting incipient cytorrhysis (%)†				Appressorium formation (%)‡	
	1 m	2 m	3 m	4 m	Hydrophobic	Hydrophilic
Guy11	30.7 ± 5.0	52.0 ± 5.7	67.0 ± 7.1	83.3 ± 4.2	99.1 ± 2.2	0
∆*Moimd4*	42.0 ± 3.2*	65.0 ± 2.0*	80.0 ± 2.1*	92.0 ± 2.6*	98.5 ± 2.8	0
MoImd4	34.7 ± 4.2	56.0 ± 5.5	72.0 ± 1.5	79.4 ± 3.2	99.5 ± 3.2	0

^†^Different concentrations of glycerol (1 to 4 m) were used to analyse incipient cytorrhysis. At least 100 appressoria were counted for each concentration.

^‡^Appressorium formation on hydrophilic or hydrophobic surfaces at 24 h post incubation; ±SD was calculated from three repeated experiments and asterisks indicate statistically significant differences (Duncan’s new multiple range test, *means *P* < 0.01).

### MoImd4 is important for XMP biosynthesis

**Figure 3:**
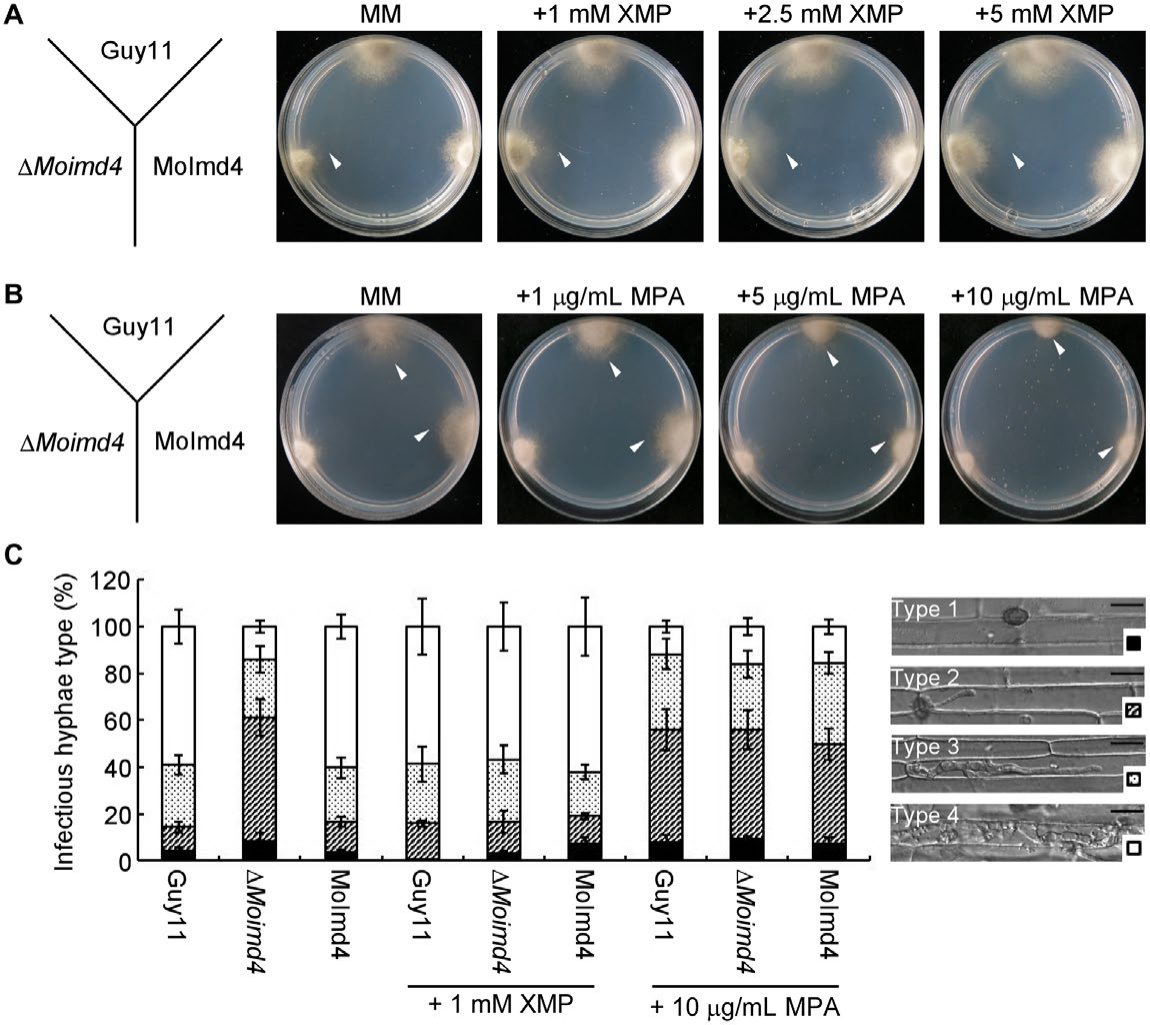
Xanthosine monophosphate (XMP) levels are critical for the growth and virulence of *Magnaporthe* *oryzae*. (A) Guy11, ∆*Moimd4* and the complemented strains were co‐incubated on minimal medium (MM) treated with or without 1, 2.5 and 5 mM XMP at 28 °C in the dark for 7 days. White arrowheads represent the edges of mycelia. (B) The indicated strains were co‐incubated on MM with or without 1, 5 and 10 µg/mL mycophenolic acid (MPA) at 28 °C for 7 days in the dark. White arrowheads represent the edges of mycelia. (C) Four types of IHs in rice sheath cells; the four different shapes are shown in the bottom right‐hand corner: type 1, no penetration; type 2, a single invasive hypha; type 3, extensive hyphal growth in only one rice cell; type 4, extensive hyphal growth in neighbouring rice cells. Statistical analysis of the type of IH of the indicated strains, treated with or without 1 mM XMP or 10 µg/mL MPA at 48 h; approximately 100 IHs were counted and the experiments were repeated three times with similar results. The error bars indicate the standard deviations of three replicates. Bar, 10 μm.

In *S. cerevisiae*, IMPDH catalyses the hydrolysis of IMP to XMP (Hyle *et al.*, 2003). To test whether the ∆*Moimd4* mutant failed to form XMP contributing to the defects in vegetative growth, conidial formation and virulence, we measured the intracellular level of XMP by high‐performance liquid chromatography (HPLC) and found that the ∆*Moimd4* mutant showed a 0.15‐fold reduction compared with the wild‐type and complemented strains (Fig. S4A, see Supporting Information). Consistent with this result, the addition of 1, 2.5 and 5 mM XMP in MM restored the defects of the mutant, with the exception of conidial formation (Figs 3A, S4B and Table 3). To further test virulence, we injected a conidial suspension amended with 1 mM XMP into detached rice sheaths. We found that, in Guy11 and the complemented strain, nearly 80% of invasion hyphae (IHs) were type 4 and type 3, with 20% showing type 1 and type 2. Exogenous addition of 1 mM XMP restored the expansion of IHs of the ∆*Moimd4* mutant to near the levels of the wild‐type and complemented strain (80% of type 3 and type 4 IHs). This was in contrast with that of the ∆*Moimd4* mutant which normally produced 60% type 2 IHs and 40% type 3 and type 4 IHs (Fig. 3C). The results indicate that the hydrolytic activity of MoImd4 and XMP levels are critical for the pathogenicity of *M. oryzae*.

**Table 3:** Phenotype analysis of the wild type, ∆*Moimd4* mutant, the complement strains, point mutation mutants and three CBS domain deletion mutants.

Strain	Colony diameter (CM)*	Colony diameter (MM)*	Colony diameter (MM + XMP)†	Conidiation (×100/cm^2^)‡	Conidiation + XMP (×100/cm^2^)§
Guy11	4.8 ± 0.1^A^	2.9 ± 0.1^A^	3.0 ± 0.2^A^	741.9 ± 50.9^A^	746.3 ± 73.1^A^
∆*Moimd4*	3.1 ± 0.1^EF^	0.9 ± 0.1^D^	2.8 ± 0.1^A^	27.9 ± 2.5^C^	25.1 ± 4.1^C^
T268AR269A	3.0 ± 0.1^FG^	1.3 ± 0.1^C^	2.3 ± 0.3^B^	29.3 ± 6.4^C^	30.7 ± 3.8^C^
S345AC347A	2.0 ± 0.1^H^	1.4 ± 0.1^B^	2.7 ± 0.2^A^	46.3 ± 7.3^C^	55.1 ± 5.0^C^
D380AG382A	3.3 ± 0.2^E^	0.8 ± 0.1^D^	1.3 ± 0.1^C^	41.9 ± 4.5^C^	33.1 ± 8.2^C^
G403A	3.2 ± 0.1^EF^	0.8 ± 0.1^D^	0.8 ± 0.1^D^	46.8 ± 4.1^C^	43.9 ± 7.6^C^
R458AY459A	3.9 ± 0.1^C^	0.4 ± 0.1^E^	0.4 ± 0.1^E^	33.0 ± 3.6^C^	27.9 ± 6.1^C^
Y428A	3.7 ± 0.1^D^	0.3 ± 0.1^E^	0.5 ± 0.1^DE^	21.6 ± 3.8^C^	21.8 ± 5.4^C^
∆CBS1	4.5 ± 0.2^B^	3.0 ± 0.2^A^	3.0 ± 0.1^A^	684.2 ± 40.1^AB^	624.5 ± 50.2^B^
∆CBS2	4.5 ± 0.1^B^	2.9 ± 0.2^A^	3.0 ± 0.2^A^	624.3 ± 32.2^B^	630.2 ± 35.1^B^
∆CBS1CBS2	4.1 ± 0.1^C^	0.4 ± 0.1^E^	0.4 ± 0.1^E^	33.7 ± 2.1^C^	29.9 ± 6.5^C^
MoImd4	4.8 ± 0.2^A^	2.9 ± 0.1^A^	2.9 ± 0.2^A^	726.3 ± 32.2^A^	746.3 ± 15.0^A^

^*^Colony diameter of the indicated strains on CM or MM after 7 days of incubation at 28 °C; ±SD was calculated from three repeated experiments and different letters indicate statistically significant differences (Duncan’s new multiple range test: *P* < 0.01).

^†^Colony diameter of the indicated strains on MM with added 1 mM XMP after 7 days of incubation at 28 °C; ±SD was calculated from three repeated experiments and letters indicate statistically significant differences (Duncan’s new multiple range test: *P* < 0.01).

^‡^Quantification of the conidial production of the indicated strains from SDC cultures in the dark for 7 days, followed by incubation under constant illumination for 3 days at room temperature; ±SD was calculated from three repeated experiments and different letters indicate statistically significant differences (Duncan’s new multiple range test: *P* < 0.01).

^§^Quantification of the conidial production of the indicated strains from SDC cultures with added 1 mM XMP in conidial suspension in the dark for 7 days, followed by incubation under constant illumination for 3 days at room temperature; ±SD was calculated from three repeated experiments and different letters indicate statistically significant differences (Duncan’s new multiple range test, letters mean *P* < 0.01).

### Impaired XMP biosynthesis results in attenuated growth and virulence

In humans, mycophenolic acid (MPA) is an approved drug to target IMPDH as a method of immunosuppressive and antiviral chemotherapy (Chapuis *et al.*, 2000; Gollapalli *et al.*, 2010; Johnson *et al.*, 2013; Morrow *et al.*, 2012; Umejiego *et al.*, 2004). Interestingly, 5 and 10 µg/mL of MPA induced the growth defect of the wild‐type and the complemented strains in MM, similar to that of the ∆*Moimd4* mutant (Figs 3B, S4C). Further infection assay in rice sheaths showed that the IHs of Guy11 and the complemented strain were mostly of type 2 when 10 µg/mL of MPA was added to the conidial suspensions, similar to those of the ∆*Moimd4 *mutant (Fig. 3C). These results indicate that MPA specifically inhibits MoImd4 and attenuates the growth and virulence of *M. oryzae*.

### Inactivation of MPA binding sites attenuates MoImd4 activity

**Figure 4:**
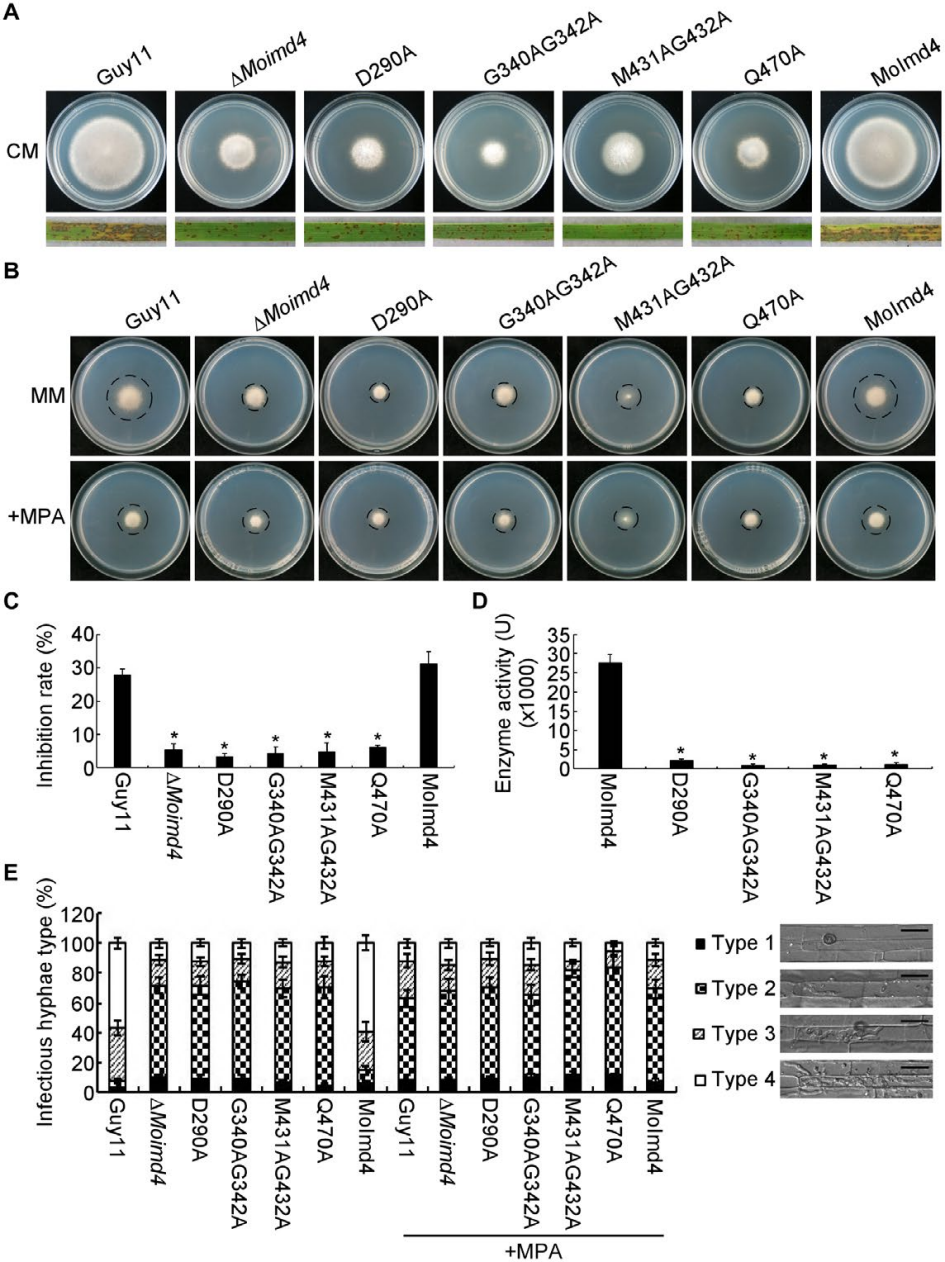
Inactivation of mycophenolic acid (MPA) binding sites leads to attenuation of MoImd4 activity. (A) Vegetative growth of Guy11, ∆*Moimd4*, D290A, G340AG342A, M431AG432A and Q470A mutants, and the complemented strains, on complete medium (CM) at 7 days in the dark. Conidial suspensions (5 × 10^4^ spores/mL) of the indicated strains were sprayed onto 11‐day‐old rice seedlings. Photographs were taken at 7 days post‐inoculation (dpi). (B) Vegetative growth of Guy11, ∆*Moimd4*, D290A, G340AG342A, M431AG432A, Q470A and the complemented strains on minimal medium (MM) treated with or without 10 µg/mL MPA. (C) Inhibition rates of the indicated strains on MM treated with or without 10 µg/mL MPA. Experiments were repeated three times with similar results. The error bars indicate the standard deviation of three replicates. Asterisks indicate statistically significant differences (Duncan’s new multiple range test, *P* < 0.01). (D) Detection of enzymatic activities of the indicated strains *in vitro*. Target proteins were expressed in *Escherichia coli* BL21‐CodonPlus (DE3) cells. We defined the production of 1 mM xanthosine monophosphate (XMP) per milligram of protein per minute as 1 U of enzyme activity. Experiments were repeated three times with similar results. The error bars indicate the standard deviations of three replicates. Asterisks indicate statistically significant differences (Duncan’s new multiple range test, *P* < 0.01). (E) Statistical analysis of IHs of the indicated strains with or without 10 µg/mL MPA at 48 h post‐inoculation (hpi); approximately 100 IHs were counted and the experiments were repeated three times. The error bars indicate the standard deviations of three replicates. Asterisks indicate statistically significant differences (Duncan’s new multiple range test, *P* < 0.01). The four types of grading standard are shown, as in Fig. 3C. Bar, 10 μm.

To further explore the function of MoImd4 in the XMP biosynthesis pathway, we modelled the three‐dimensional structure of MoImd4 using the structure of CnImd1 (PDB entry 4af0.2.A), with which it shares 65.19% amino acid identity as the template (Arnold *et al.*, 2006; Benkert *et al.*, 2011; Biasini *et al.*, 2014). MoImd4 forms a tetramer and has conserved potential MPA binding sites at D290, G340, G342, M431, G432 and Q470 (Fig. S5A,B, see Supporting Information). We then generated four point mutation mutants [∆*Moimd4/MoIMD4* ^D290A ^(D290A), ∆*Moimd4/MoIMD4* ^G340AG342A^ (G340AG342A), ∆*Moimd4/MoIMD4* ^M431AG432A^ (M431AG432A) and ∆*Moimd4/MoIMD4* ^Q470A^ (Q470A)] and tested their functions (Fig. S6, see Supporting Information). These mutants presented similar phenotypic defects in vegetative growth on CM and virulence to the ∆*Moimd4 *mutant (Fig. 4A). The vegetative growth and invasion assays in rice cell tests of these point mutation mutants treated with or without exogenous MPA (10 µg/mL) were consistent with the fact that MPA cannot bind with these allelic mutants of MoImd4 (Fig. 4B,C,E). Moreover, all of these mutant proteins showed weakened Imd enzymatic activities of approximately 0.04‐fold compared with that of the wild‐type (Fig. 4D).

### Functional characterization of different domains and reaction sites of MoImd4

**Figure 5:**
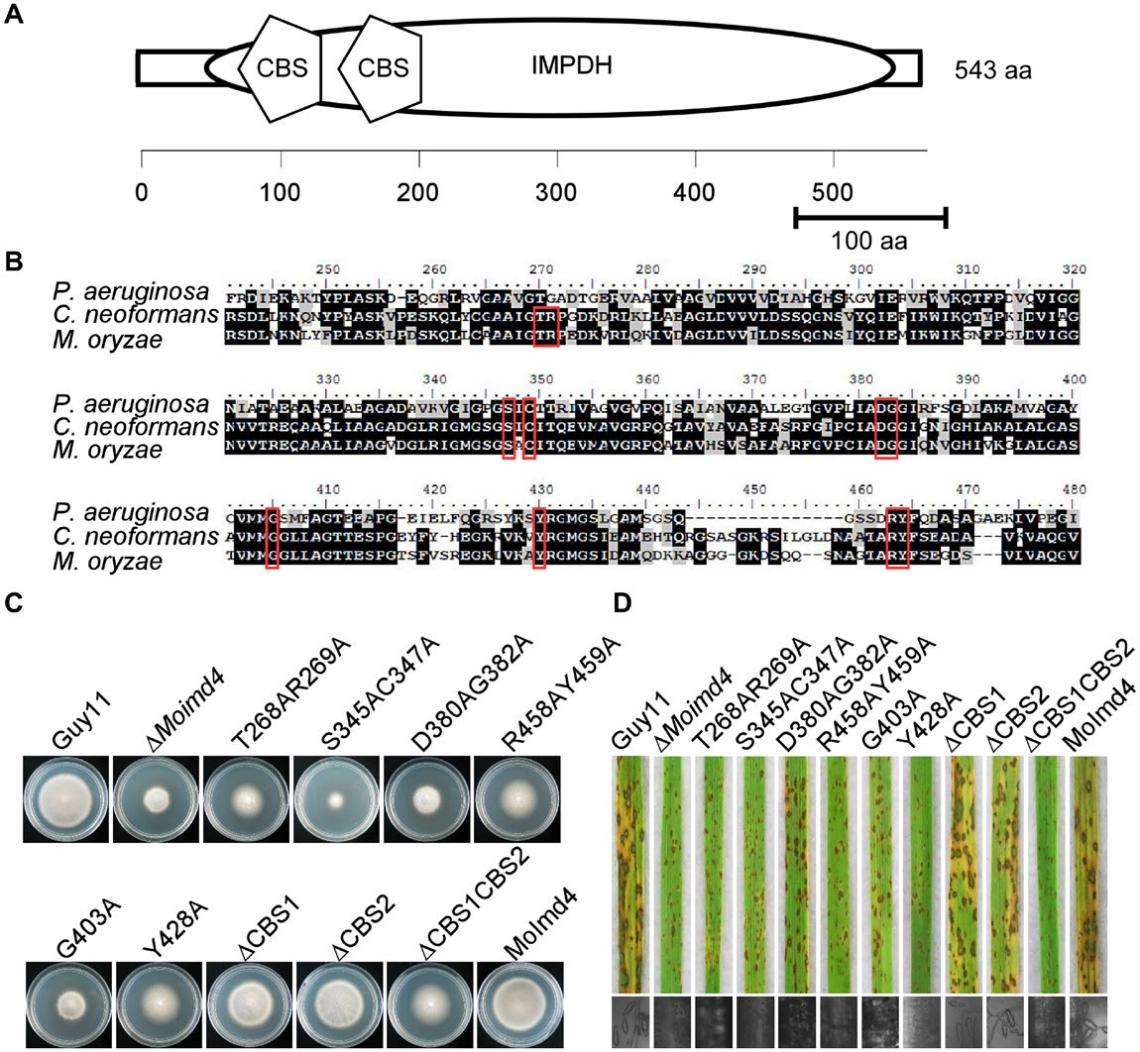
Functional characterization of cystathionine β‐synthase (CBS) domains and reaction sites of MoImd4. (A) Schematic representation of MoImd4 inosine‐5′‐monophosphate dehydrogenase (IMPDH) domain (oval) and tandem CBS subdomains (pentagon). Domains were predicted using the SMART program (http://smart.embl-heidelberg.de/). aa, amino acid. (B) Multiple alignments of *Pseudomonas* *aeruginosa*, *Cryptococcus* *neoformans* and *Magnaporthe* *oryzae *IMPDH proteins. Red boxes represent conservative functional sites involved in the interaction with the substrates. The amino acid identity of IMPDH was 37% between *M. oryzae* and *P. aeruginosa*, and 63% between *M. oryzae* and *C. neoformans*. (C) The wild‐type Guy11, ∆*Moimd4*, the complemented strains, predicted site mutation mutants and CBS domain deletion mutants were incubated on complete (CM) at 28 °C in the dark and photographed after 7 days of incubation. (D) Pathogenicity test on rice seedlings of the indicated strains. Infected rice leaves were illuminated under fluorescent light for 24 h to produce conidia. The lesions were observed under a light microscope.

MoImd4 has one conserved IMPDH domain (amino acids 47–533) and two tandem accessory CBS subdomains (CBS1, amino acids 136–187; CBS2, amino acids 199–247) (Fig. 5A). Previous studies have shown that sites such as S317, C319, D358, G360, G381, Y405, R418 and Y419 of *Pseudomonas aeruginosa* are required for its catalytic function (Rao *et al.*, 2013). Based on the IMPDH of *C. neoformans* and *P. aeruginosa*, we predicted similar sites in MoImd4 (Fig. 5B), and generated six point mutation mutants: ∆*Moimd4/MoIMD4* ^T268AR269A ^(T268AR269A), ∆*Moimd4/MoIMD4* ^S345AC347A ^(S345AC347A), ∆*Moimd4/MoIMD4* ^D380AG382A ^(D380AG382A), ∆*Moimd4/MoIMD4* ^R458AY459A ^(R458AY459A), ∆*Moimd4/MoIMD4* ^G403A^ (G403A) and ∆*Moimd4/MoIMD4* ^Y428A^ (Y428A). We also generated three CBS domain deletion mutants: ∆*Moimd4/MoIMD4* ^∆CBS1 ^(∆CBS1), ∆*Moimd4/MoIMD4* ^∆CBS2 ^(∆CBS2) and ∆*Moimd4/MoIMD4* ^∆CBS1CBS2 ^(∆CBS1CBS2) (Fig. S6). All of these mutant alleles, except ∆CBS1 and ∆CBS2, exhibited defects in vegetative growth, conidial formation and virulence (Fig. 5C,D and Table 3). These results demonstrate that the tandem CBS domain and the reaction sites are important for MoImd4 function.

### MoImd4 is important in the purine metabolic pathway

**Figure 6:**
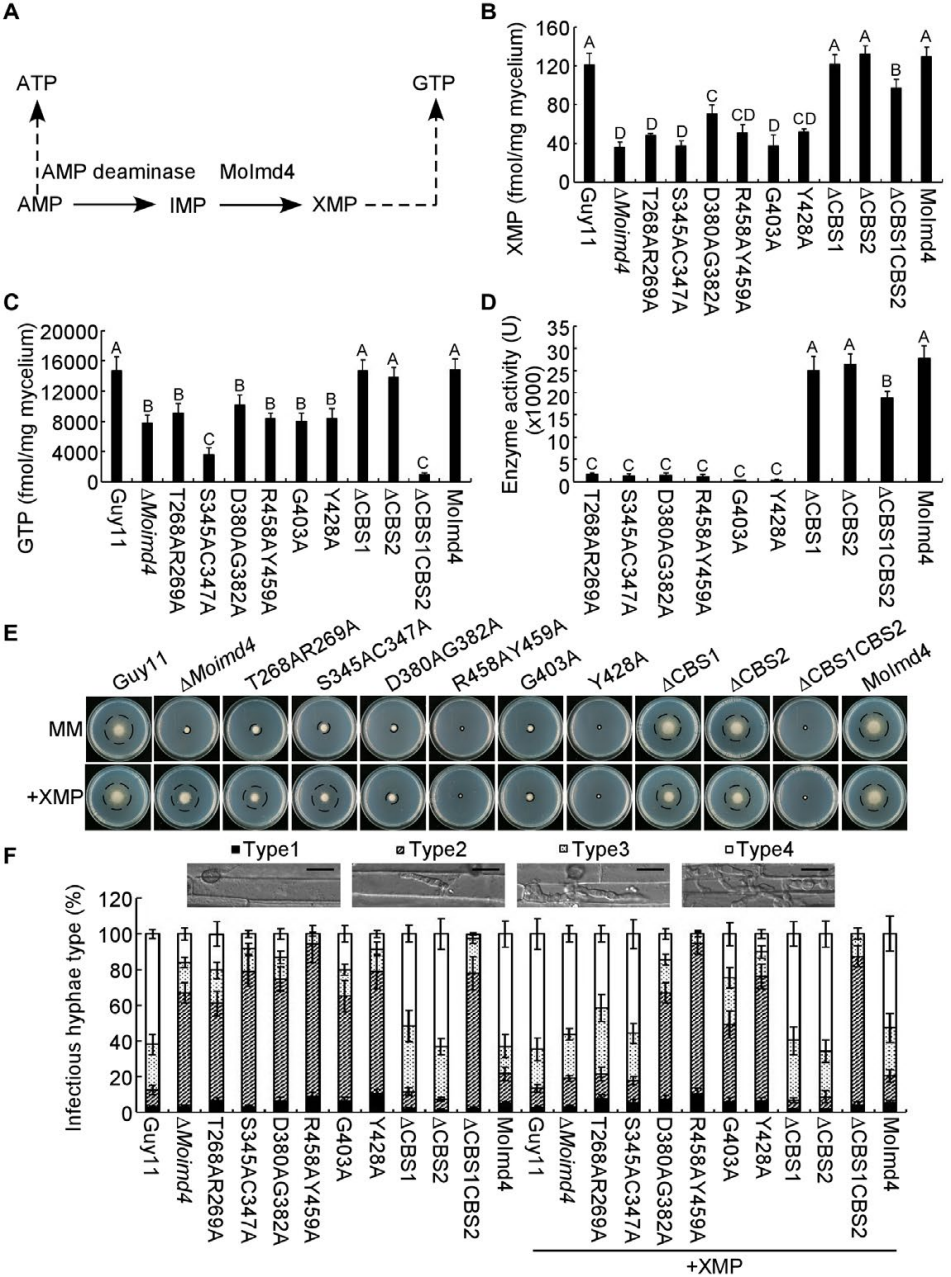
MoImd4 is required for the purine metabolic pathway in *Magnaporthe oryzae *and exogenous xanthosine monophosphate (XMP) suppresses the defects of the S345AC347A mutant in vegetative growth and virulence. (A) The *de novo* GTP/ATP biosynthesis pathway of *M. oryzae*. (B, C) Intracellular levels of XMP/GTP in mycelia of the indicated strains by high‐performance liquid chromatography (HPLC). Experiments were repeated three times with similar results. The error bars indicate the standard deviations of three replicates. Different letters indicate statistically significant differences (Duncan’s new multiple range test, *P* < 0.01). (D) Detection of enzymatic activities of the indicated strains *in vitro*. The target proteins were expressed in *Escherichia coli* BL21‐CodonPlus (DE3) cells. Experiments were repeated three times with similar results. The error bars indicate the standard deviation of three replicates. Different letters indicate statistically significant differences (Duncan’s new multiple range test, *P* < 0.01). (E) Vegetative growth and statistical analysis of the indicated strains on minimal medium (MM) treated with or without 1 mM XMP after 7 days of incubation. Experiments were repeated three times with similar results. (F) Statistical analysis of the type of IHs of the indicated strains with or without 1 mM XMP at 48 h post‐inoculation. Approximately 100 IHs were counted and experiments were repeated three times. The error bars indicate the standard deviations of three replicates. Please refer to Fig. 3C for IH grading. Bar, 10 μm.

The purine metabolic pathway provides ATP and GTP essential for cellular processes and activities (Morrow *et al.*, 2012). To evaluate the role of MoImd4 in the *de novo* purine biosynthesis pathway, we measured *in vivo *intracellular XMP and GTP levels in the ∆*Moimd4* mutant, point mutation strains, CBS domain deletion mutants, wild‐type and complemented strains (Fig. 6A). The XMP levels were reduced to 0.33‐fold in the ∆*Moimd4* mutant and all six point mutation mutants, and to 0.83‐fold in the tandem CBS deletion mutants, when compared with the control strains. However, there was little difference between the solely CBS domain deletion mutants and the wild‐type (Fig. 6B). We also found that GTP levels were remarkably reduced in most of the mutant strains in comparison with Guy11 and complemented strains, with especially low levels of 0.2‐fold and 0.06‐fold found in S345AC347A and ∆CBS1CBS2 strains, respectively. However, the ∆CBS1 and ∆CBS2 mutants showed no attenuation (Fig. 6C). Moreover, we purified the point mutation proteins by His‐tag and examined the enzymatic activities of these point mutation proteins and domain deletion proteins. The IMPDH activity was nearly attenuated in these point mutation mutants (Fig. 6D), despite the fact that these mutations did not affect the three‐dimensional structure (Fig. S7, see Supporting Information). MoImd4 enzyme activity was reduced in ∆CBS1CBS2, but not in any of the CBS deletion mutants (Fig. 6D).

### Exogenous XMP suppresses defects in vegetative growth and virulence of the S345AC347A mutant

To further understand which sites are critical for the function of MoImd4 in mediating XMP synthesis, XMP was added to MM. Only the S345AC347A point mutation mutant rescued the defects in vegetative growth and pathogenicity. However, the T268AR269A mutant was partially restored in vegetative growth (0.8‐fold) and the formation of type 4 IHs up to 0.4‐fold (0.6‐fold when compared with the ∆*Moimd4* and S345AC347A mutants). None of the other mutants exhibited a similar rescue of the defect (Fig. 6E,F and Table 3). It should be noted that, similar to the ∆*Moimd4 *mutant, none of these strains was rescued in conidial formation by exogenous XMP (Table 3). Together, these results show that MoImd4 governs the production of XMP and that S345 and C347 sites are the most critical for this activity.

### MoImd4 interacts with MoPdeH to promote its phosphodiesterase activity

Owing to the interaction between MoPdeH and MoImd4, we hypothesized that MoImd4 affects the phosphodiesterase activity of MoPdeH. To test this, we first expressed GST‐MoPdeH and His‐MoImd4 fusion proteins and verified this interaction using glutathione‐*S*‐transferase (GST) pull‐down assays (Fig. S8A, see Supporting Information). Then, we measured the MoPdeH phosphodiesterase activity with and without the presence of MoImd4 using purified proteins *in vitro *and a fluorescence‐based assay method for free phosphate according to Yin *et al.* (2018). Our results showed that the samples treated with MoPdeH exhibited strong fluorescence, whereas samples treated with various concentrations of MoImd4 showed more intense fluorescence (Fig. S8B), suggesting that MoImd4 could promote the phosphodiesterase activity of MoPdeH *in vitro*. Meanwhile, we also measured intracellular cAMP levels in Guy11, ∆*Moimd4* and ∆*MopdeH* mutants, and found that cAMP levels in the ∆*Moimd4* mutant were 2.0‐fold higher than in Guy11, but 0.5‐fold lower than in the ∆*MopdeH* mutant (Fig. S8C), indicating that MoImd4 promotes the phosphodiesterase activity of MoPdeH.

### T268, R269, D380, G382, R458, Y459, G403 and Y428 of MoImd4 are important in promoting MoPdeH phosphodiesterase activity

**Figure 7:**
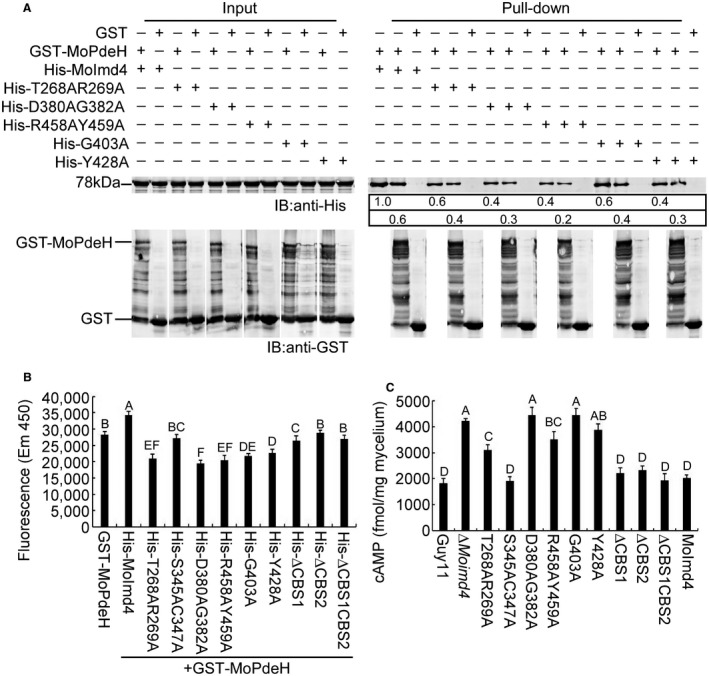
T268, R269, D380, G382, R458, Y459, G403 and Y428 of MoImd4 are important for the promotion of MoPdeH enzymatic activity. (A) Glutathione‐*S*‐transferase (GST) pull‐down assays for interactions between GST‐MoPdeH and His‐MoImd4, His‐T268AR269A, His‐D380AG382A, His‐R458AY459A, His‐G403A and His‐Y428A alleles of MoImd4. Equal amounts of MoPdeH‐GST or GST protein were incubated with Glutathione Sepharose beads for 3 h at 4 °C prior to mixing with His infusion protein lysates for another 3 h at 4 °C. Equal His‐tag infusion proteins from input were used as controls. The eluted proteins were detected by western blot analysis with anti‐His and anti‐GST antibodies. The number represents the intensity of eluted proteins detected by the anti‐His antibody (top panel, elution protein; bottom panel, elution protein after the same dilution). The intensity of the elutions from wild‐type MoImd4 was set to 1.0. (B) Purified His‐fusion expression proteins affecting the enzyme activity of MoPdeH. Equal amounts of His‐fusion proteins and GST‐MoPdeH were mixed to measure the enzymatic activities. Fluorescence was read by a 10‐min kinetic reaction with excitation at 420 nm and emission at 450 nm. Experiments were repeated three times with similar results. The error bars indicate the standard deviations of three replicates. Different letters indicate statistically significant differences (Duncan’s new multiple range test, *P* < 0.01). (C) Intracellular cyclic adenosine monophosphate (cAMP) level assay of the indicated strains at the mycelial stage. Experiments were repeated three times with similar results. The error bars represent ± standard deviation (SD) of three replicates. Different letters indicate statistically significant differences (Duncan’s new multiple range test, *P* < 0.01).

As some of the point mutation mutants did not restore the defects in vegetative growth or pathogenicity of the ∆*Moimd4 *mutant by exogenous XMP (Fig. 6E,F), and we have evidence that MoImd4 promotes MoPdeH enzymatic activity, we hypothesized that some of these residues may play a role in the interaction with MoPdeH. We used GST pull‐down assays to determine that T268A, R269A, D380A, G382A, G403A, Y428A, R458A and Y459A of MoImd4 attenuate the interaction between MoImd4 and MoPdeH (Fig. 7A). We also measured the enzyme activities of MoPdeH in the presence of MoImd4 with various point mutations. MoImd4 promoted the enzyme activity of MoPdeH, whereas S345AC347A, ∆CBS2 and ∆CBS1CBS2 had no effect, and T268AR269A, D380AG382A, G403A, Y428A and R458AY459A had only certain effects (Fig. 7B). We next detected the cAMP levels of Guy11, the ∆*Moimd4* mutant, various point mutation mutants of *MoIMD4*, CBS domain deletion mutants and the complemented strains *in vivo*. Intriguingly, cAMP levels of the S345AC347A mutant and three CBS domain deletion mutants were similar to those of Guy11 and the complemented strains. However, the cAMP levels of T268AR269A and R458AY459A mutants were significantly different from those of the ∆*Moimd4* mutant, and were higher than those of Guy11 and the complemented strains. Meanwhile, the cAMP levels of the D380AG382A, G403A and Y428A mutants were close to those of the ∆*Moimd4* mutant and were about 2.0‐fold higher than those of Guy11 (Fig. 7C). Taken together, these results provide direct evidence that T268, R269, D380, G382, G403, Y428, R458 and Y459 of MoImd4 are important in promoting the enzyme activity of MoPdeH.

### MoPdeH and MoImd4 show mutual regulations of their enzymatic activities

**Figure 8:**
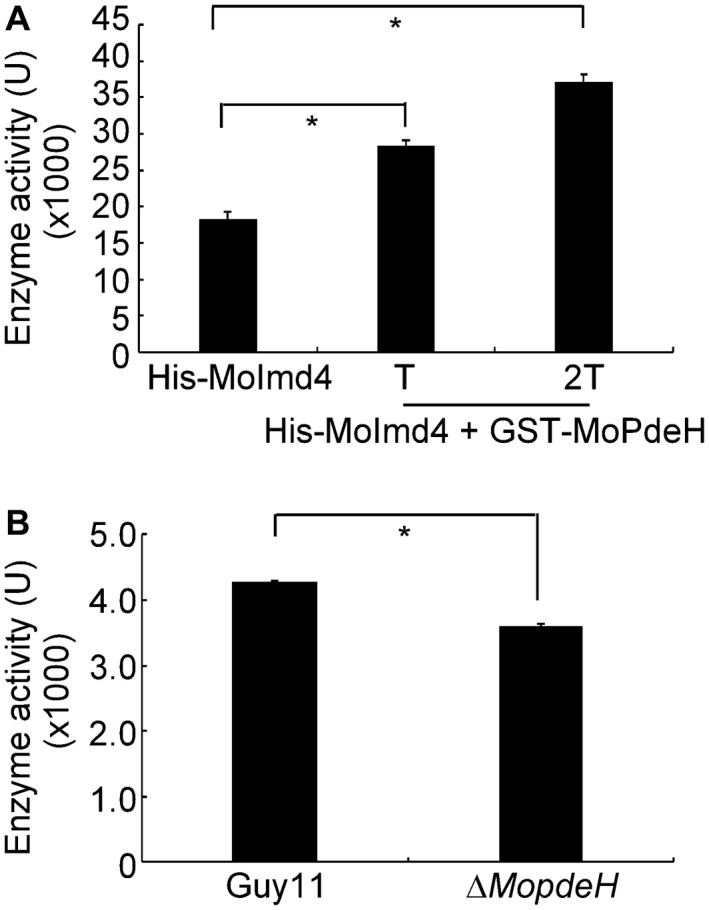
MoPdeH promotes the enzymatic activity of MoImd4. (A) Enzymatic activity of purified His‐MoImd4 proteins, with 1 and 2 T of the purified GST‐MoPdeH protein. The production of xanthosine monophosphate (XMP) was monitored by the absorbance at 290 nm. ‘T’ represents the total quantity of the His‐MoImd4 protein. Experiments were repeated three times with similar results. The error bars indicate the standard deviation of three replicates. Asterisks indicate statistically significant differences (Duncan’s new multiple range test, *P* < 0.01). (B)* In vivo*, we extracted total protein from the wild‐type Guy11 and ∆*MopdeH* as the crude enzyme of MoImd4, and then added it to the enzymatic reaction system for 5 min; the production of XMP was monitored by the absorbance at 290 nm. Experiments were repeated three times with similar results. The error bars indicate the standard deviation of three replicates. Asterisks indicate statistically significant differences (Duncan’s new multiple range test, *P* < 0.01).

MoImd4 interacts with MoPdeH to promote its phosphodiesterase activity; however, the function of MoPdeH on the interaction with MoImd4 remains unclear. We purified the GST‐MoPdeH and His‐MoImd4 proteins *in vitro* and tested the enzymatic activity of MoImd4. The results showed that MoImd4 activity without treatment was approximately 18 kU, but increased continuously when purified exogenous MoPdeH protein was added (Fig. 8A). We also extracted total proteins from the wild‐type Guy11 and ∆*MopdeH* strains as the crude enzyme of MoImd4, and added them to the enzymatic reaction system. The result showed that the enzyme activity of MoImd4 in the Guy11 strain was significantly higher than that in the ∆*MopdeH* mutant, indicating that MoPdeH also promotes the enzymatic activity of MoImd4.

## Discussion

In eukaryotic cells, the G‐protein/cAMP‐dependent signalling pathway is involved in the sensing of extracellular signals and integrating them into intrinsic pathways (Malbon, 2005). cAMP acts as a second messenger to transmit extracellular hormones and nutrients into the intracellular environment, where it activates downstream targets (Daniel *et al.*, 1998). In the rice blast fungus *M. oryzae, *high‐affinity phosphodiesterase MoPdeH exhibits various regulatory functions in hyphal autolysis, spore morphology, CWI and pathogenicity, as well as surface signal recognition (Ramanujam and Naqvi, 2010; Yang *et al.*, 2017; Zhang *et al.*, 2011aa). To understand the underlying mechanisms, we searched for proteins that interact with MoPdeH, and identified MoImd4. We characterized MoImd4 as an IMPDH in the purine synthetic pathway and found that MoImd4 functions in the growth and pathogenicity of the fungus. We also revealed that MoImd4 interacts with MoPdeH to impact the development and pathogenicity of *M. oryzae *collectively.

*MoIMD4* gene disruption leads to the formation of atypical and restricted lesions in rice leaves, which is interesting, as IMPDH is not known to directly affect fungal virulence. We speculated that it could involve the following mechanisms: (i) the loss of MoImd4 severely impacts fungal growth and fitness levels; (ii) the loss of MoImd4 may induce host‐derived defence which restricts infection. Generally speaking, the first layer of broad‐spectrum defence against any pathogen is met by conserved pathogen‐associated molecular patterns (PAMPs) in the host that trigger PAMP‐triggered immunity (PTI). However, 3,3′‐diaminobenzidine (DAB) staining of rice sheaths following infection showed no significant difference in reactive oxygen species (ROS) levels between the ∆*Moimd4* mutant and wild‐type (Fig. S9, see Supporting Information). As MoImd4 is located in the cytoplasm than secreted into the host cell during infection, and regulates the conversion of IMP to XMP affecting the *de novo* purine metabolic pathway (Fig. S10, see Supporting Information), we considered that MoImd4 may function intracellularly to affect the pathogenicity of *M. oryzae*. Given that the defect in growth and invasion in rice cells of the ∆*Moimd4* mutant can be suppressed by the addition of exogenous XMP, it is plausible that the uptake of XMP may be critical for *M. oryzae*. In accordance with this reasoning, the virulence defect exhibited by the mutants with amino acid variants or purines could be supplemented by adding relevant exogenous amino acids and purines, including *ILV2/6*, *LYS2/20*,* STR3/MET6/MET13*, *CPA2* and *ADE1 *(Chen *et al.*, 2014; Fernandez *et al.*, 2013; Liu *et al.*, 2016a; Rao *et al.*, 2013; Saint‐Macary *et al.*, 2015; Wilson *et al.*, 2012; Yan *et al.*, 2013; Zhang *et al.*, 2014).

Tandem CBS subdomains of IMPDH exist extensively in eukaryotic organisms. In this study, we discovered that two CBS domains had overlapping functions in development, and tandem CBS deletion mutants caused defects in growth, conidial formation and pathogenicity. The enzymatic activity of the MoImd4 ∆CBS1CBS2 allele and XMP contents were statistically significantly reduced, but the levels of GTP were also sharply reduced. This is consistent with the study of the *E. coli* *guaB* ^ΔCBS^ mutant (GuaB homologous to MoImd4) (Pimkin and Markham, 2008). The three‐dimensional protein structure of GuaB IMPDH was a tetramer with tandem CBS domains in each monomer (Pimkin and Markham, 2008). Homology modelling revealed that the tandem CBS domains were not present in the protein model owing to disorder in *M. oryzae*, similar to *C. neoformans *and *P. aeruginosa* (Morrow *et al.*, 2012; Rao *et al.*, 2013). However, in *A. gossypii*, the regulatory CBS pair domains of IMPDH form octamers with GDP and GTP, resulting in decreased affinity between the catalytic domain and substrate IMP (Buey *et al.*, 2015b, 2017). In consequence, tandem CBS domains take part in *de novo* purine metabolism and play an essential role in GTP level control. Nevertheless, it was debatable whether the content of GTP and the location of CBS subdomains in protein structures were different in *M. oryzae* than in other organisms.

**Figure 9:**
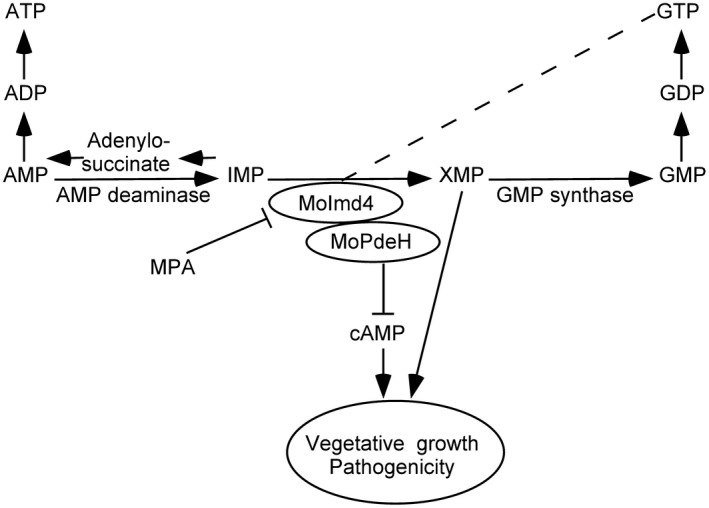
A proposed model for crosstalk between the *de novo* purine metabolic pathway and the intracellular cyclic adenosine monophosphate (cAMP) signalling pathway in *Magnaporthe* *oryzae*. Evidence supports a novel crosstalk pathway between *de novo* purine metabolism and cAMP signalling through MoPdeH and xanthosine monophosphate (XMP) regulating synergistically the growth and virulence of *M. oryzae*. GMP, guanosine monophosphate; IMP, inosine‐5′‐monophosphate; MPA, mycophenolic acid.

Our results revealed, for the first time, that MoPdeH interacts with MoImd4. In our study, sequence alignments of MoImd4 revealed several conserved amino acid sites whose mutations exhibited three different situations: (i) S345AC347A mutation affects the enzymatic activity of MoImd4, but not the interaction between MoImd4 and MoPdeH, and the phosphodiesterase activity of MoPdeH; yet the phenotypic defect is restored with exogenous XMP; this is similar to the recovery of the ∆*Moimd4* mutant phenotype with XMP; (ii) T268AR269A mutation affects the enzymatic activity of both MoImd4 and MoPdeH, and the intensity of the interaction between them, and exogenous XMP partially rescues the defects in growth and pathogenicity; (iii) D380A, G382A, R458A, Y459A, G403A and Y428A mutations all affect the enzymatic activities of MoImd4 and MoPdeH, and the intensity of interaction between them, but exogenous XMP has no effect on their phenotypes. We surmised that the reasons for this might derive from the manner of interaction between MoPdeH and MoImd4. We found that treatment with MPA or inactivation of the MPA binding sites of MoImd4 produced no differences in the interaction between MoImd4 and MoPdeH (Fig. S11A,B, see Supporting Information). Further, inactivation of the MPA binding sites did not affect the phosphodiesterase activity of MoPdeH, which is similar to that of the S345AC347A mutation (Fig. S11C). Based on these results, we speculated that, when certain sites are changed, such as T268, R269, D380, G382, R458, Y459, G403 and Y428, they interfere with the interaction between MoPdeH and MoImd4, attenuating MoPdeH enzymatic activity, indicating that the interaction between MoImd4 and MoPdeH is important for the enzymatic activity of MoPdeH. Based on this reasoning, we propose that MoImd4 mediates crosstalk between the cAMP pathway and the *de novo* purine biosynthesis pathway, which influence the vegetative development and virulence of *M. oryzae *collectively (Fig. 9).

Why is purine biosynthesis linked to cAMP regulation? Previous work has demonstrated that, when the cytosolic pH value is maintained around neutral, adenylate cyclase is activated by increasing the affinity of the enzyme for ATP, which induces cAMP accumulation (Orij *et al.*, 2011; Purwin *et al.*, 1986). In eukaryotic cells, ATP/GTP is involved in various cellular biological processes, including signal transduction, gene transcription and cellular respiration (Ganapathy‐Kanniappan and Geschwind, 2013; Koopman *et al.*, 2012; Pathak *et al.*, 2013). The levels of ATP/GTP are maintained by the purine nucleotide pool sizes, including transcriptional control and enzyme‐level regulation of purine biosynthetic enzymes. Enzyme‐level regulation works as the ‘first line of defence’ to rapidly balance specific fluxes in purine biosynthesis (Petersen, 1999; Yamaoka *et al*., 1996; Zalkin and Nygaard, 1996). Here, we found that MoImd4, which functions as the rate‐limiting and first committed step in the *de novo* biosynthesis of GTP, controls not only the synthesis of ATP/GTP, but also the balance between them *in vivo*. Thus, we speculate that the synthesis and hydrolysis of intracellular cAMP may require energy that comes from the *de novo* purine biosynthesis system.

MPA is a specific IMPDH inhibitor that perturbs the *de novo* purine metabolic pathway (Johnson *et al.*, 2013; Kohler *et al.*, 2005; Umejiego *et al.*, 2004; Wei *et al.*, 2016). Treatment with MPA attenuates the growth and virulence of *M. oryzae *and further assays confirm that the inhibition constant is significantly reduced following mutations of MoImd4 in D290, G340, G342, M431, G432 and Q470 (Table 4). However, as IMPDH mediation of *de novo* purine biosynthesis is highly conserved, questions emerge as to whether the IMPDH gene(s) in rice will also be affected by MPA. BLAST alignments found that there was 42% amino acid sequence identity between MoImd4 and LOC_Os03g56800.1 encoding rice IMPDH (OsIMPDH). Homology modelling analysis showed that OsIMPDH could also form a tetramer (Fig. S12, see Supporting Information). However, differences, including kinetic profiles, were found between these two proteins (Table 4). Thus, although the possibility exists, MPA may exert more influence on MoImd4 than on OsIMPDH*.* This is consistent with studies of *Candida* *albicans* and *C. neoformans*, in which *K*_m_ values for both IMP and NAD were substantially different from those of human forms, despite sharing high amino acid sequence identities (Kohler *et al.*, 2005; Morrow *et al.*, 2012). Our additional studies showed that MPA did not affect appressorium formation or host invasion (Table S2, see Supporting Information).

**Table 4:** Kinetic parameters of IMPDHs from *M. oryzae* and rice.

Parameter	*K*_m_(IMP) (μM)	*K*_m_(NAD) (μM)	*K*_ii_(NAD) (μM)
MoImd4	216.2 ± 56.4	857.8 ± 186.9	3.3
D290A	142.7 ± 15.3	304.9 ± 143.5	1.4
G340AG342A	91.8 ± 15.7	156.1 ± 76.2	1.7
M431AG432A	79.4 ± 12.2	293.8 ± 86.1	2.2
Q470A	151.9 ± 21.9	63.1 ± 7.8	1.1
OsIMPDH	76.5 ± 23.2	193.7 ± 98.2	0.4

Steady‐state and inhibition constants for inosine‐5’‐monophosphate dehydrogenase (IMPDH) from *Magnaporthe oryzae* and the rice.

In summary, our results reveal that MoImd4 plays a critical role in growth, development and virulence of *M. oryzae* by mediating the *de novo* purine biosynthesis pathway. MoImd4 promotes the enzymatic activity of MoPdeH to influence intracellular cAMP levels. Our results provide new insights into how cAMP signalling and purine metabolism collectively regulate the growth and pathogenicity of *M. oryzae.*

## Experimental Procedures

### Strains and cultures

Guy11 was used as the wild‐type in this work. All strains were cultured on CM and MM at 28 °C in the dark. Mycelia were harvested from strains grown in liquid CM for 48 h for DNA and RNA extraction. Protoplasts were prepared and transformed as described previously (Sweigard *et al.*, 1992). Transformants were selected on TB3 medium (3 g yeast extract, 3 g casamino acids, 200 g sucrose and 7.5 g agar in 1 L distilled water) with 300 µg/mL hygromycin B (Roche) or 200 µg/mL zeocin (Invitrogen, Carlsbad, CA, USA). For conidial formation, mycelial blocks were incubated on SDC medium at 28 °C for 7 days in the dark, followed by 3 days of continuous illumination under fluorescent light.

### Targeted gene deletion assay

The *MoIMD4* deletion mutant was generated using the standard one‐step gene replacement strategy. The gene deletion vector was constructed by polymerase chain reaction (PCR) amplification using two 1.0‐kb sequences flanking the targeted gene and the primer pairs given in Table S3 (see Supporting Information). The resulting PCR products were digested with restriction endonucleases and ligated with the hygromycin resistance cassette (*HPH*) released from pCX62. Candidate mutants were first screened by PCR and later confirmed by Southern blot analysis.

### Generation of the complemented, point mutation and CBS‐deficient green fluorescent protein (GFP) or His‐fusion constructs

In order to generate GFP fusion constructs, target genes containing the native promoter of *MoIMD4* were amplified by PCR (Table S3) and inserted into the pYF11 plasmid (bleomycin resistance) using the yeast gap repair approach (Bruno *et al.*, 2004). The plasmids were verified by sequencing prior to being introduced into the protoplasts of the ∆*Moimd4* mutant. For D290, G340, G342, M431, G432, Q470, T268, R269, S345, C347, D380, G382, G403, Y428, R458 and Y459 point mutation vector construction, we introduced alanine to specifically inactivate the sites, and imminent adjacent residues to generate double point mutations, for example G340AG342A, M431AG432A, T268AR269A, S345AC347A and R458AY459A. CBS deletion mutants were generated using a similar approach to that described above. The zeocin‐resistant transformants were confirmed by the presence of GFP signals and by western blot analysis. To generate His‐fusion constructs, cDNA was amplified by PCR using Phanta^®^ Super‐Fidelity DNA Polymerase (Vazyme Biotech Co., Ltd., Nanjing, China) and cloned into the pET‐32a vector for expression in *E. coli *(BL21‐CodonPlus).

### Vegetative growth assays

For vegetative growth, mycelial blocks were cut from the edge of 5‐day‐old cultures and placed onto medium in the dark at 28 °C for 7 days (Zhang *et al.*, 2010) prior to evaluation. To test the effect of exogenous XMP, strains were cultured on MM supplemented with XMP at final concentrations of 1, 2.5 and 5 mM. For inhibitor assays, strains were inoculated on MM treated with 1, 5 and 10 µg/mL MPA in the dark at 28 °C for 7 days.

### Conidial and appressorial formation, turgor pressure, pathogenicity and rice sheath penetration assays

Conidia were induced by culture on SDC medium in the dark at 28 °C for 7 days, followed by constant illumination for 3 days at room temperature. Conidia were collected and 1 × 10^5^ spores/mL were used for appressorium formation. More than 200 appressoria were counted for each strain at 24 h post‐inoculation (hpi), and the experiments were repeated at least three times. For appressorium turgor assays, 1–4 m glycerol solution was used to measure the collapse of appressoria, as described previously (Liu *et al.*, 2016bb).

For spray assays, conidia were collected and adjusted to 5 × 10^4^ spores/mL in a 0.2% (w/v) gelatin solution. Eleven‐day‐old rice seedlings (*Oryza sativa *cv. CO‐39) were sprayed and incubated in a chamber at 28 °C with 90% humidity in the dark for the first 24 h, followed by a 12‐h/12‐h light/dark cycle for 7 days. The rice sheath penetration assays were carried out as described previously (Liu *et al.*, 2016bb). XMP or MPA was added to the conidial suspension and hyphal expansion was assessed at 48 hpi.

### Pull‐down assays

GST, GST‐MoPdeH, His‐MoImd4, His‐T268AR269A, His‐D380AG382A, His‐R458AY459A, His‐G403A and His‐Y428A were expressed in *E. coli *BL21‐CodonPlus (DE3) cells (Stratagene, Cedar Creek, TX, USA) and proteins were induced as described previously (Yang *et al.*, 2017; Yin *et al.*, 2016). Supernatants of lysed cells of purified GST‐MoPdeH or GST proteins were incubated with 30 µL of Glutathione Sepharose™ 4B (GE Healthcare, Lot 10148479, Sweden) at 4 °C for 3 h, followed by centrifugation (500 g, 2 min). Lysed His‐infusion proteins were re‐incubated with Glutathione Sepharose™ beads at 4 °C for another 3 h. Finally, the beads were washed with washing buffer [20 mM Tris (pH 7.5), 0.25 mM NaCl, 2 mM ethylenediaminetetraacetic acid (EDTA), 2 mM ethyleneglycolbis(b‐aminoethylether)‐N,N′‐tetraacetic acid (EGTA)] five times prior to elution with elution buffer [20 mM Tris (pH 7.5), 0.25 mM NaCl, 2 mM EDTA, 2 mM EGTA, 1 mM reduced glutathione, pH 8.0]. Eluted proteins were analysed by western blotting with anti‐His and anti‐GST antibodies.

### Measurements of IMPDH *in vitro* activity, and *K*_m_ and *K*_ii_ constants

His, His‐MoImd4, His‐T268AR269A, His‐S345AC347A, His‐D380AG382A, His‐R458AY459A, His‐G403A, His‐Y428A, His‐D290A, His‐G340AG342A, His‐M431AG432A, His‐Q470A, His‐∆CBS1, His‐∆CBS2, His‐∆CBS1CBS2 and GST‐MoPdeH were expressed and purified with Ni‐NTA agarose (Qiagen, Lot 151047819, Germany) and Glutathione Sepharose™ 4B (GE Healthcare, Lot 10148479). Enzyme activities were estimated in 100 mM Tris‐HCl, pH 8.1, 10 mM KCl, 0.1 mM dithiothreitol (DTT) in the presence of 250 µM NAD (Solarbio, Lot 427D0213) and 500 µM IMP (Absin, abs42019840, Shanghai, China), and the purified proteins of His and His‐MoImd4 with or without equal protein of GST‐MoPdeH for 5 min. The production of XMP was monitored by the absorbance at 290 nm (Pimkin and Markham, 2008) in a 96‐well cell culture plate (CELLTER, CS016‐0096). A standard curve of absorbance vs. concentration was established using commercial xanthosine‐5′‐monophosphate disodium (Alading, X113495). The amount of purified His‐fusion protein was kept the same. Protein concentrations were estimated using a standard Bradford protein assay kit (Beyotime, P0006). For *K*_m_ value estimation, IMP and NAD concentrations were adjusted over a range from 100 to 8000 µM, with 250 µM NAD and 500 µM IMP, respectively (Morrow *et al.*, 2012). When one substrate remains the same and the other changes over different concentrations, the reaction velocity equals the linear slope of XMP increase by a 10‐min kinetic reaction (Green *et al.*, 2012). Non‐linear fitting of the data to the Michaelis–Menton equation (Equation 1) and the uncompetitive substrate inhibition equation (Equation 2) was performed by Origin 8.0:

(1) $$V=V_{\text{max}}\left[\text{S}\right]/\left(K_{\text{m}}+\left[\text{S}\right]\right)$$

(2) $$\text{1}/V=\left(\text{1}+\left[\text{I}\right]/K_{\text{ii}}\right)/V_{\text{max}}+K_{\text{m}}/V_{\text{max}}\left[\text{S}\right]$$

where *V *is the initial velocity, *V*_max_ is the maximum velocity, *K*_m_ is the Michaelis constant, [S] is the substrate concentration, [I] is the inhibitor concentration and *K*_ii_ is the inhibitor constant.

### Intracellular XMP, GTP and cAMP measurements

The indicated strains were cultured on CM and a certain amount of mycelium was grown in liquid CM for 2 days. Then, the mycelium was pressed dry and quickly ground into a powder in liquid nitrogen before lyophilizing for 24 h. Each mycelium sample was treated following previously established procedures (Liu *et al.*, 2016b). Samples were quantified by HPLC analysis as described previously (Liu *et al.*, 2016b). The peak flow of the standard of XMP (Alading, X113495) was at ~6.4 min, of the standard of GTP (Santa Cruz, sc‐203062, USA) was at ~2.6 min, and of the standard of cAMP (Meilune, MB3159) was at ~13.8 min. The measurements of XMP, GTP and cAMP were performed according to the method reported previously (Liu *et al.*, 2016b).

### Effects of MoImd4 and point mutation proteins on *in vitro* MoPdeH phosphodiesterase activitiy

GST‐MoPdeH, His‐MoImd4, His‐T268AR269A, His‐D380AG382A, His‐R458AY459A, His‐G403A, His‐Y428A, His‐D290A, His‐G340AG342A, His‐M431AG432A, His‐Q470A and His were induced and purified as described previously (Yang *et al.*, 2017). The phosphodiesterase activity was detected by fluorescence intensity. The N‐[2‐(1‐maleimidyl)ethyl]‐7‐(diethylamino)coumarin‐3‐carboxamide (MDCC) fluorophore coupled with phosphate and the fluorescence of the phosphate sensor increase approximately six‐ to eight‐fold, which can be measured in real time (Brune *et al.*, 1994). The reaction was performed as follows: 5 µM cAMP (Meilune, MB3159), purified GST‐MoPdeH with or without equal purified His‐fusion proteins, separately, and 10 mU/mL alkaline phosphatase (Calbiochem, 524545, San Diego, CA, USA) were incubated in PDE enzymatic reaction buffer (50 mM Tris, pH 7.6, 100 mM NaCl, 10 mM MgCl_2_, 0.01% Triton^®^ X‐100 and 0.5 mM DTT) for 60 min at room temperature, and 0.5 µM phosphate sensor (Invitrogen, PV4406) in phosphate sensor detection buffer (20 mM Tris, pH 7.6 and 0.05% Triton^®^ X‐100) was added to each well. The plate was mixed and read by a 10‐min kinetic reaction with excitation at 420 nm and emission at 450 nm. Positive and negative controls were included as described previously (Yang *et al.*, 2017). The experiments were repeated three times and each experiment had three replicates.

### Homology modelling assay

The amino acid sequences of MoImd4 and OsIMPDH were submitted to the online platform SWISS‐MODEL (https://www.swissmodel.expasy.org/interactive/xBVTUG/templates/) to predict the protein structures, and the structures were analysed by PyMOL.

## Supporting information


**Fig. S1  **Southern blot analysis of the *MoIMD4* deletion mutant. (A) The strategy of *MoIMD4* gene replacement. Fragments of the *MoIMD4* coding region were replaced with hygromycin phosphotransferase (*HPH*) fragments. (B) Southern blot analysis of the *MoIMD4* knockout mutant with specific probe (probe 1) and *HPH* probe (probe 2).

**Fig. S2  **Phylogenetic analysis of MoImd4 and other inosine monophosphate dehydrogenase (Imd) proteins by CLUSTAL_W and MEGA 6 programs. Species names and accession numbers are as follows:* M. oryzae *(*Magnaporthe oryzae* XP_003716182.1), *M. robertsii *(*Metarhizium robertsii *XP_007816906.1),* C. purpurea *(*Claviceps purpurea *CCE29804.1),* T. reesei *(*Trichoderma reesei *XP_006966877.1),* C. orbiculare *(*Colletotrichum orbiculare *ENH80323.1),* G. tritici *(*Gaeumannomyces tritici* XP_009224448.1),* S. cerevisiae *(*Saccharomyces cerevisiae* NP_013536.3),* C. neoformans *(*Cryptococcus neoformans* XP_012046718.1),* H. sapiens *(*Homo sapiens* NP_000875.2),* P. aeruginosa *(*Pseudomonas aeruginosa* WP_070336446.1),* B. anthracis *(*Bacillus anthracis* WP_047401536.1),* S. pyogenes *(*Streptococcus pyogenes *WP_002991454) and* T. foetus* (*Tritrichomonas foetus* OHT09030.1).

**Fig. S3  **MoImd4 is involved in vegetative growth. (A) The wild‐type Guy11, ∆*Moimd4* and the complemented strains were cultured on complete medium (CM), minimal medium (MM), straw decoction and corn agar media (SDC) and oatmeal media (OM) at 28 °C in the dark for 7 days. (B) Mycelial pellets in liquid CM. Mycelia of Guy11, ∆*Moimd4* and the complemented strains were inoculated in liquid CM with shaking (160 rpm) for 48 h at 28 °C. Experiments were performed three times with similar results.

**Fig. S4  **MoImd4 is important for xanthosine monophosphate (XMP) biosynthesis. (A) Intracellular levels of XMP in Guy11, ∆*Moimd4* and the complemented strains estimated by high‐performance liquid chromatography (HPLC). Experiments were repeated three times with similar results. Error bars represent the standard deviations and asterisks denote statistical significances (Duncan’s new multiple range test, *P *< 0.01). (B) Statistical analysis of colony radius on minimal medium (MM) with or without XMP. Experiments were repeated three times with similar results. Error bars represent the standard deviations and asterisks denote statistical significances (Duncan’s new multiple range test, *P *< 0.01). (C) Statistical analysis of colony radius on MM with or without mycophenolic acid (MPA). Error bars represent the standard deviations and asterisks denote statistical significances (Duncan’s new multiple range test, *P* < 0.01).

**Fig. S5  **The structure of MoImd4 on binding with mycophenolic acid (MPA) and the binding sites of MPA. (A) Predicted structure of MoImd4 was a tetramer binding with MPA. Green represents the three‐dimensional structure of MoImd4; red represents the inhibitor MPA. (B) Enlarged drawing of MPA added to one of the ligands on the structure of MoImd4. Around MPA, different coloured curves predict the binding sites of MPA, including D290, G340, G342, M431, G432 and Q470.

**Fig. S6  **Western blot analysis of green fluorescent protein (GFP) expression of the point mutation and cystathionine β‐synthase (CBS)‐deficient mutants. Total proteins of the indicated strains were extracted from 2‐day‐old mycelia in liquid complete medium. Anti‐GFP was used for analysis by western blotting.

**Fig. S7  **Homology modelling of key sites and point mutants of MoImd4. The target protein sequence of MoImd4 was predicted by SWISS‐MODEL and point mutants were analysed by PyMOL. Red curves represent key sites, grey curves denote point mutants and red stars show the sites of red curves and grey curves.

**Fig. S8  **MoImd4 promotes MoPdeH phosphodiesterase activity. (A) *In vitro* glutathione‐*S*‐transferase (GST) pull‐down assays for MoImd4 and MoPdeH. The expression of GST‐MoPdeH and His‐MoImd4 was induced with 0.1 mM IPTG (isopropyl‐b‐D‐1‐thiogalactopyranoside). GST‐MoPdeH or GST was incubated with Glutathione Sepharose beads for 3 h at 4 °C, precipitated and co‐incubated with lysates containing His‐MoImd4 for an additional 3 h at 4 °C. Eluted proteins were detected by western blot analysis with anti‐His and anti‐GST antibodies. (B) Phosphodiesterase activities of purified GST‐MoPdeH proteins, with 0.1, 0.2 or 1 T of the purified His‐MoImd4 protein. Fluorescence was detected by a 10‐min kinetic reaction with excitation at 420 nm and emission at 450 nm. ‘T’ represents the total quantity of the GST‐MoPdeH protein. Experiments were repeated three times with similar results. The error bars indicate the standard deviation of three replicates. Asterisks indicate statistically significant differences (Duncan’s new multiple range test, *P *< 0.01). (C) Measurements of* in vivo* cyclic adenosine monophosphate (cAMP) levels of Guy11, ∆*Moimd4*, MoImd4, *∆MopdeH* and MoPdeH at the stage of mycelia. Experiments were repeated three times with similar results. The error bars represent ± standard deviation (SD) of three replicates. Asterisks indicate statistically significant differences (Duncan’s new multiple range test, *P *< 0.01).

**Fig. S9  **MoImd4 does not participate in reactive oxygen species (ROS) scavenging. (A) Conidial suspensions of Guy11, the ∆*Moimd4* mutant and complemented strains were injected into separate rice sheaths. At 24 h post‐inoculation (hpi), 3,3′‐diaminobenzidine (DAB) was used to dye the sheaths for 8 h. (B) Percentages of cells with infectious hyphae (IHs) were dyed with DAB. Means were calculated from three independent replicates. There were few significant differences between the strains. Bar, 10 μm.

**Fig. S10  **Locations of MoImd4‐GFP in the ∆*Moimd4* mutant at the stages of sporulation and infection.

**Fig. S11  **Interaction between MoPdeH and MoImd4 treated with mycophenolic acid (MPA). (A) Lysed cells of purified GST‐MoPdeH or glutathione‐*S*‐transferase (GST) proteins were incubated with Glutathione Sepharose™ 4B at 4 °C for 3 h with centrifugation. Then, lysed cells of purified MoImd4‐His with 1, 5 and 10 μg/mL MPA were re‐incubated with GST beads at 4 °C for another 3 h and then eluted. Equal His‐tag infusion proteins from input were used as controls. Eluted proteins were detected by western blot analysis with anti‐His and anti‐GST antibodies. The number represents the intensity of eluted proteins detected by anti‐His antibody. The intensity of the elutions from wild‐type MoImd4 was set to 1.0. (B) Lysed cells of purified GST‐MoPdeH or GST proteins were incubated with Glutathione Sepharose™ 4B at 4 °C for 3 h with centrifugation. Then, lysed cells of proteins with inactivated MPA binding sites (D290A, G340AG342A, M431AG432A and Q470A) were re‐incubated with GST beads at 4 °C for another 3 h and then eluted. Equal His‐tag infusion proteins from input were used as controls. Eluted proteins were detected by western blot analysis with anti‐His and anti‐GST antibodies. The number represents the intensity of eluted proteins detected by anti‐His antibody. The intensity of the elutions from wild‐type MoImd4 was set to unity. The S345AC347A protein was used as a control to show that mutation of these sites did not affect the interaction between MoPdeH and MoImd4. (C) Purified His‐fusion expression proteins affecting the enzyme activity of MoPdeH. Equal amounts of His‐fusion proteins and GST‐MoPdeH were mixed to measure the enzymatic activity. Fluorescence was read by a 10‐min kinetic reaction with excitation at 420 nm and emission at 450 nm. Experiments were repeated three times with similar results. The error bars indicate the standard deviations of three replicates. Asterisks indicate statistically significant differences (Duncan’s new multiple range test, *P *< 0.01).

**Fig. S12  **The predicted three‐dimensional structure of OsIMPDH.

**Table S1  **Potential interacting protein list of MoPdeH identified by yeast two‐hybrid screen.

**Table S2  **Appressorium formation on the hydrophobic surface of the wild‐type strain in the presence of mycophenolic acid (MPA).

**Table S3  **Primers used in this study.
