# Supplementary figures and images for: MoImd4 mediates crosstalk between MoPdeH‐cAMP signalling and purine metabolism to govern growth and pathogenicity in Magnaporthe oryzae
Source: Mol Plant Pathol. 2019 Jan 11;20(4):500–18. doi: 10.1111/mpp.12770 (PMC6422694; doi:10.1111/mpp.12770)

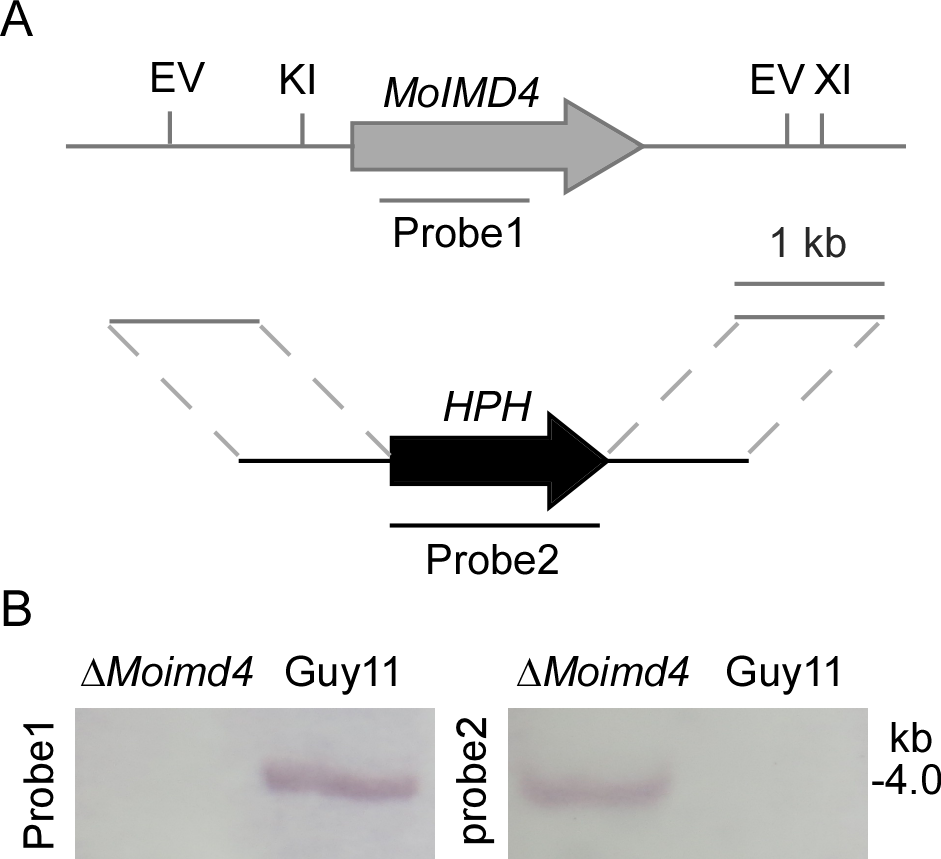

Supplement: Supplementary file 1 — Fig. S1 Southern blot analysis of the MoIMD4 deletion mutant. (A) The strategy of MoIMD4 gene replacement. Fragments of the MoIMD4 coding region were replaced with hygromycin phosphotransferase (HPH) fragments. (B) Southern blot analysis of the MoIMD4 knockout mutant with specific probe (probe 1) and HPH probe (probe 2). [file MPP-20-500-s001.tif]

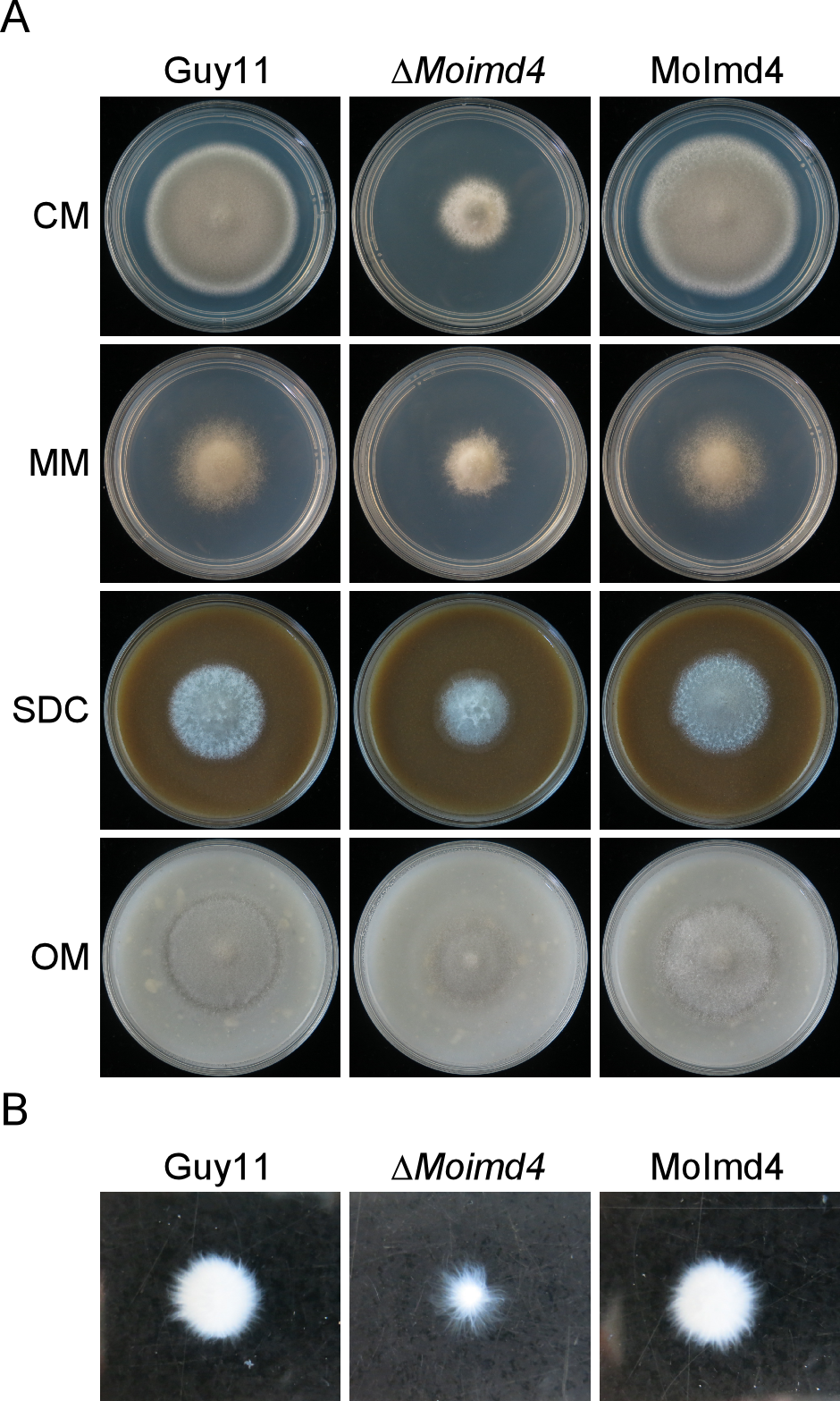

Supplement: Supplementary file 3 — Fig. S3 MoImd4 is involved in vegetative growth. (A) The wild‐type Guy11, ∆Moimd4 and the complemented strains were cultured on complete medium (CM), minimal medium (MM), straw decoction and corn agar media (SDC) and oatmeal media (OM) at 28 °C in the dark for 7 days. (B) Mycelial pellets in liquid CM. Mycelia of Guy11, ∆Moimd4 and the complemented strains were inoculated in liquid CM with shaking (160 rpm) for 48 h at 28 °C. Experiments were performed three times with similar results. [file MPP-20-500-s003.tif]

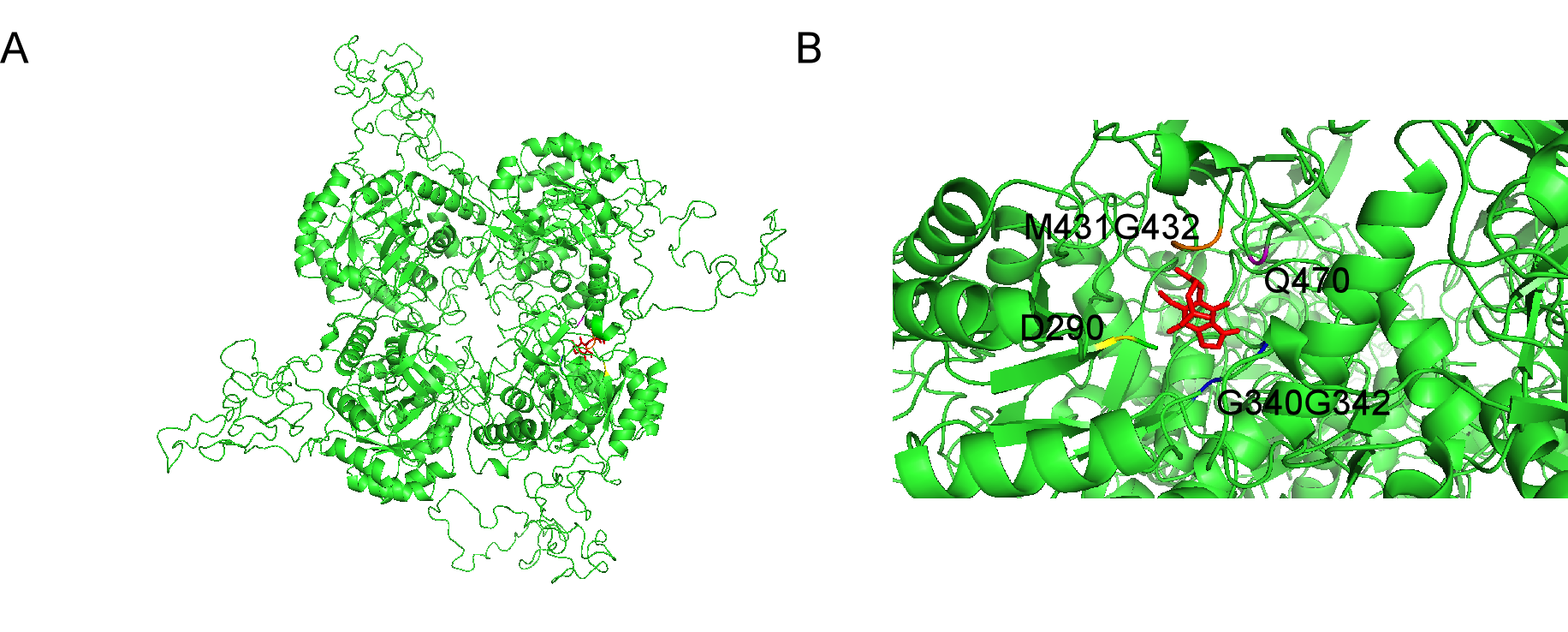

Supplement: Supplementary file 5 — Fig. S5 The structure of MoImd4 on binding with mycophenolic acid (MPA) and the binding sites of MPA. (A) Predicted structure of MoImd4 was a tetramer binding with MPA. Green represents the three‐dimensional structure of MoImd4; red represents the inhibitor MPA. (B) Enlarged drawing of MPA added to one of the ligands on the structure of MoImd4. Around MPA, different coloured curves predict the binding sites of MPA, including D290, G340, G342, M431, G432 and Q470. [file MPP-20-500-s005.tif]

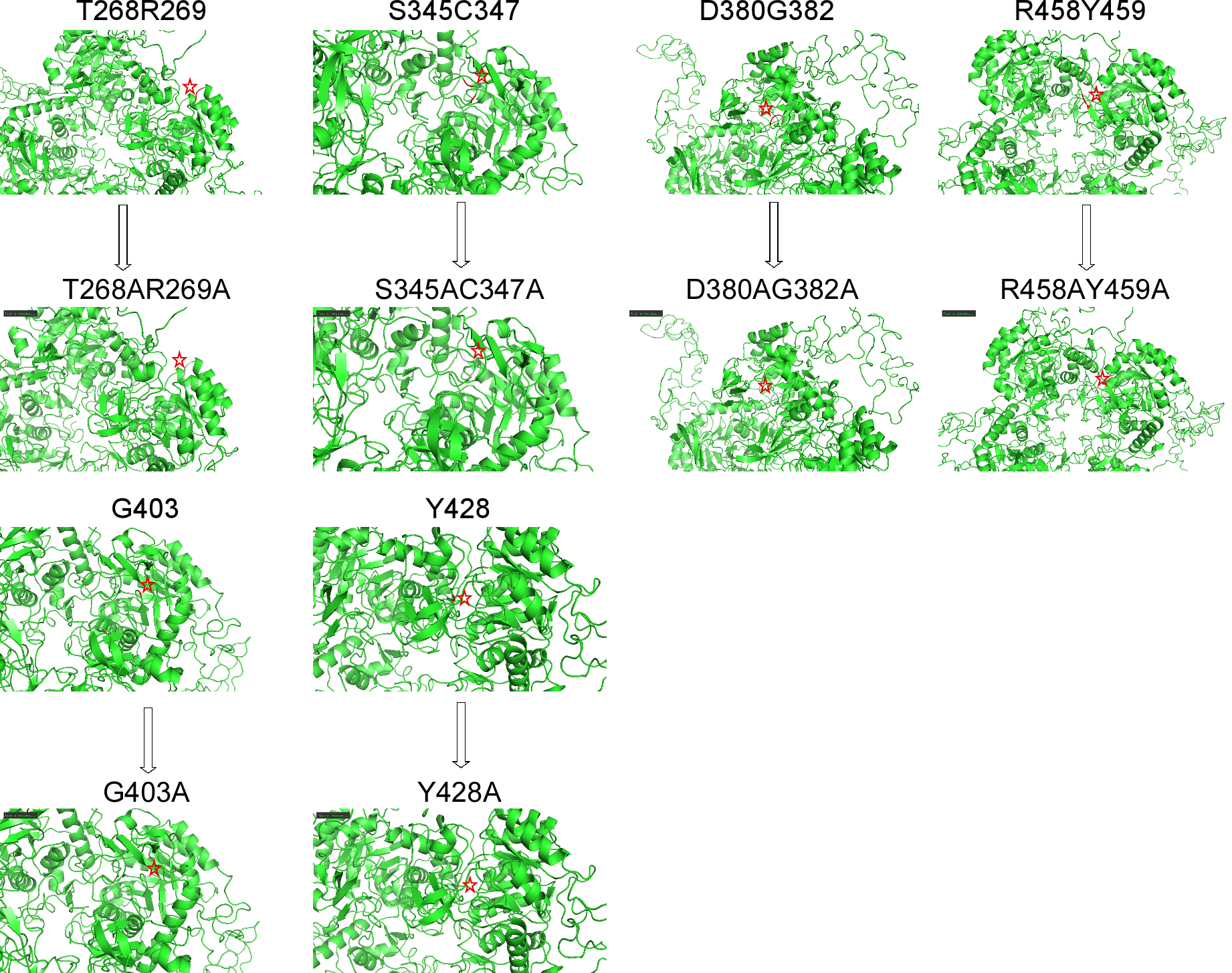

Supplement: Supplementary file 7 — Fig. S7 Homology modelling of key sites and point mutants of MoImd4. The target protein sequence of MoImd4 was predicted by SWISS‐MODEL and point mutants were analysed by PyMOL. Red curves represent key sites, grey curves denote point mutants and red stars show the sites of red curves and grey curves. [file MPP-20-500-s007.tif]

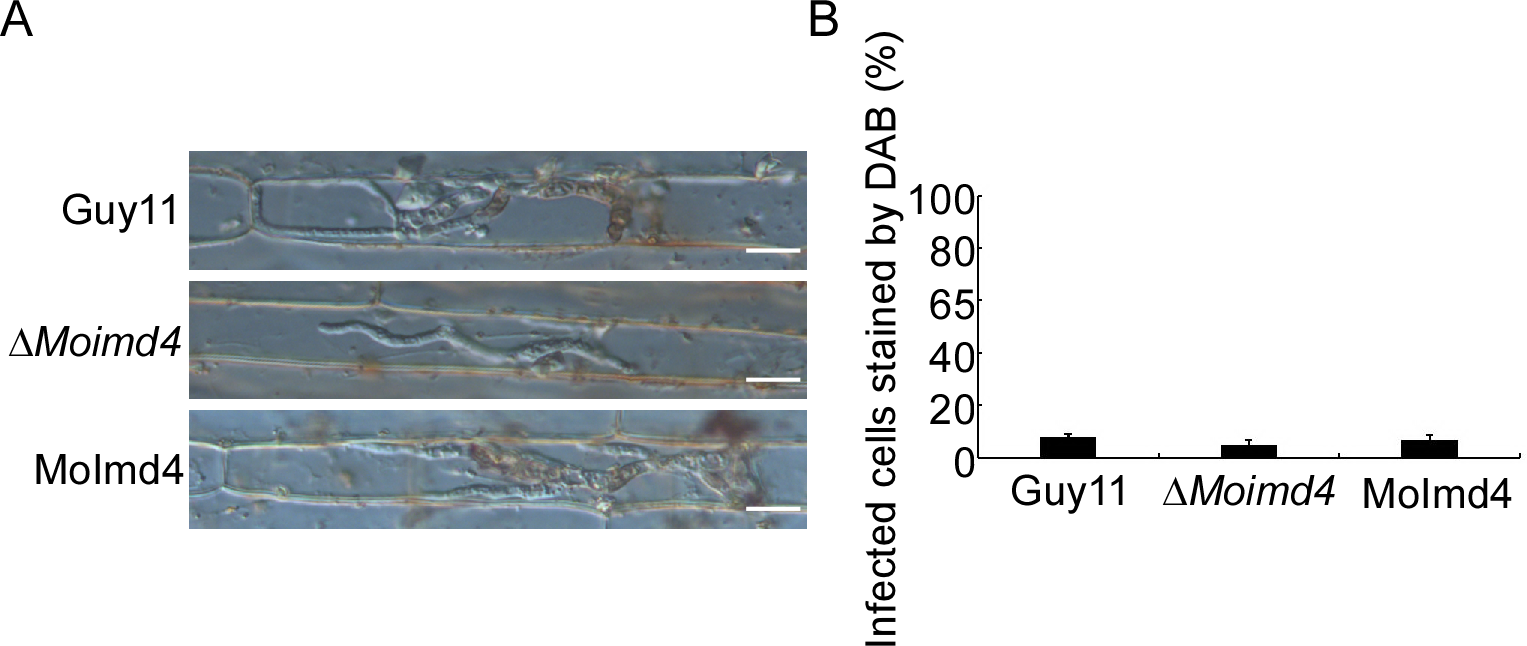

Supplement: Supplementary file 9 — Fig. S9 MoImd4 does not participate in reactive oxygen species (ROS) scavenging. (A) Conidial suspensions of Guy11, the ∆Moimd4 mutant and complemented strains were injected into separate rice sheaths. At 24 h post‐inoculation (hpi), 3,3′‐diaminobenzidine (DAB) was used to dye the sheaths for 8 h. (B) Percentages of cells with infectious hyphae (IHs) were dyed with DAB. Means were calculated from three independent replicates. There were few significant differences between the strains. Bar, 10 μm. [file MPP-20-500-s009.tif]

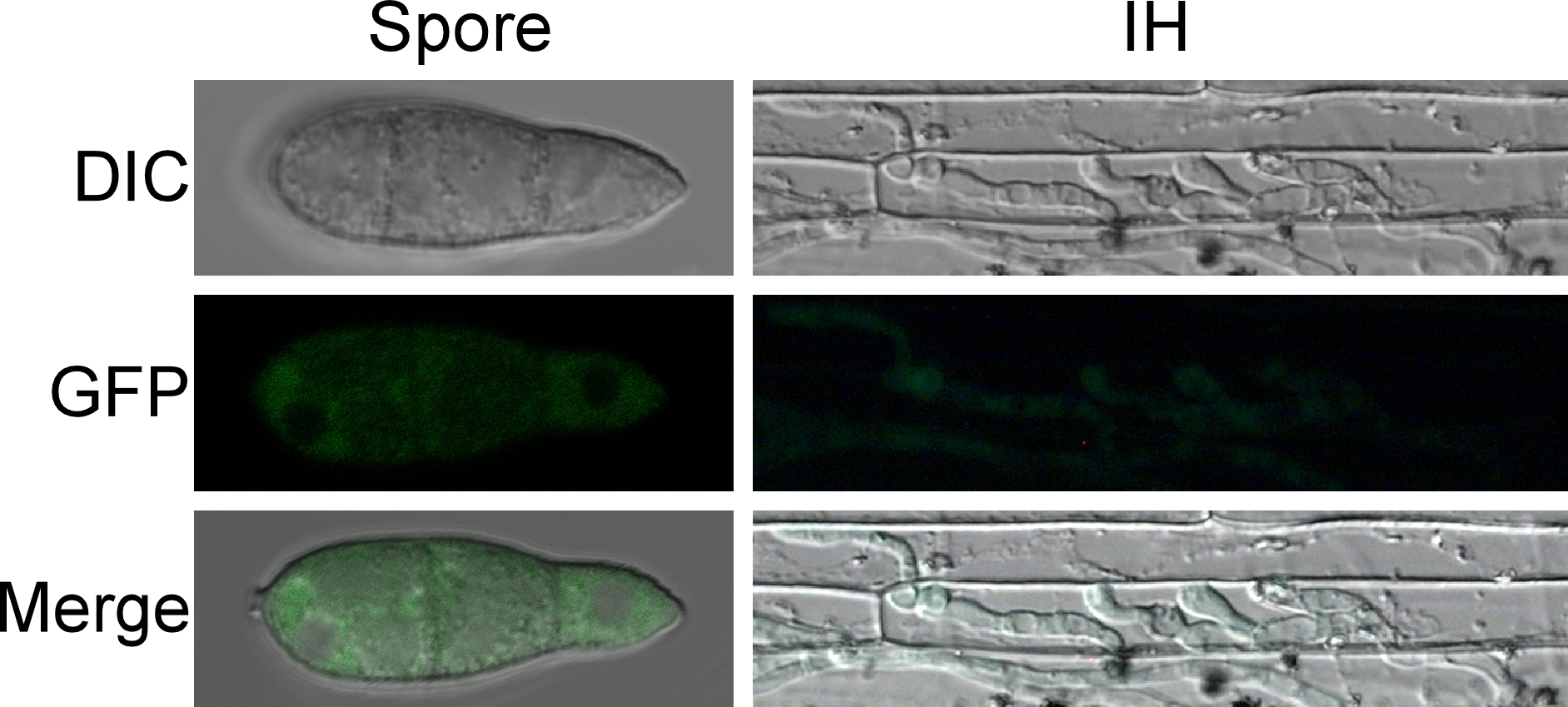

Supplement: Supplementary file 10 — Fig. S10 Locations of MoImd4‐GFP in the ∆Moimd4 mutant at the stages of sporulation and infection. [file MPP-20-500-s010.tif]

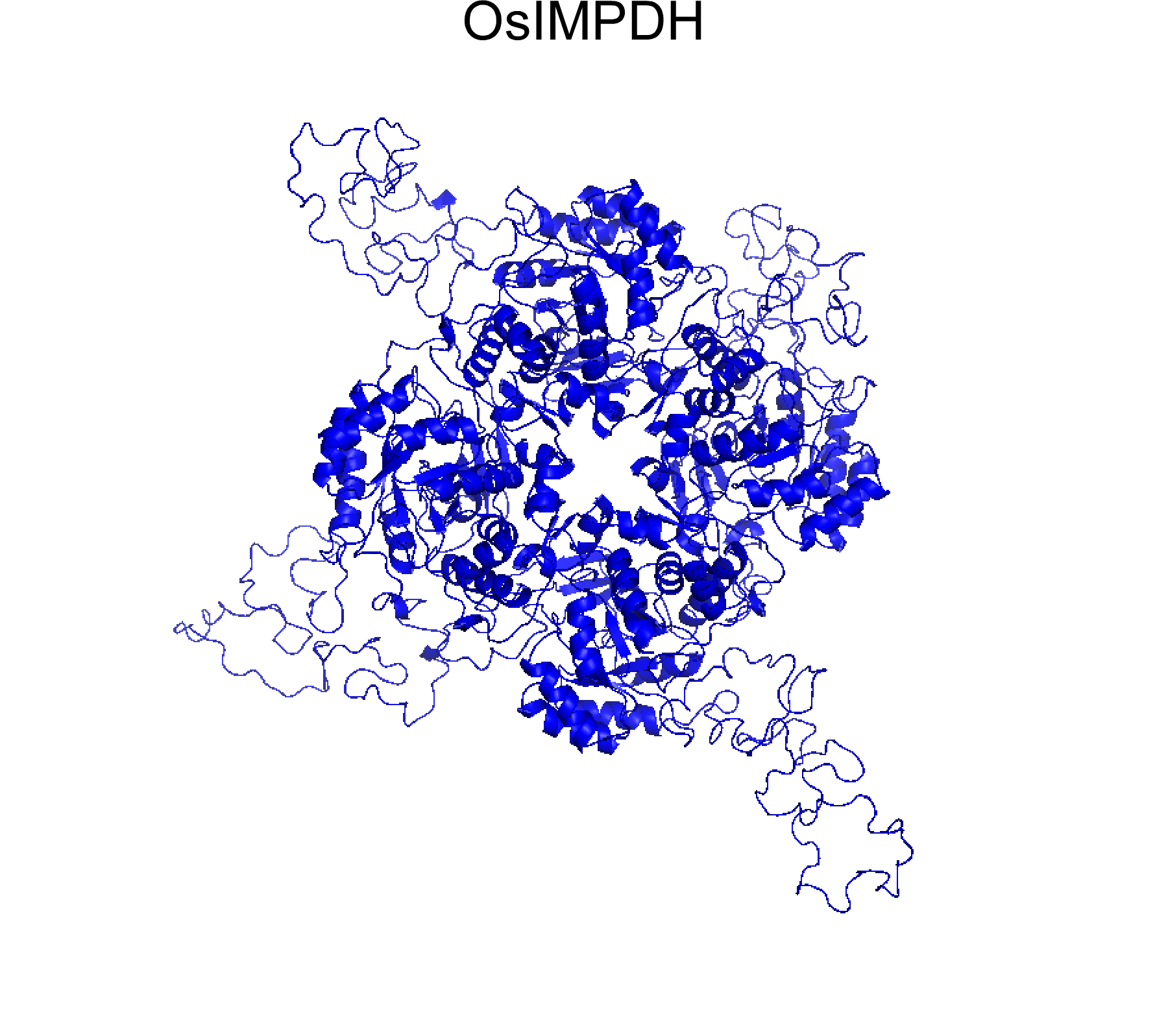

Supplement: Supplementary file 12 — Fig. S12 The predicted three‐dimensional structure of OsIMPDH. [file MPP-20-500-s012.tif]
