# Supplementary material for: MoImd4 mediates crosstalk between MoPdeH‐cAMP signalling and purine metabolism to govern growth and pathogenicity in Magnaporthe oryzae
Source: Mol Plant Pathol. 2019 Jan 11;20(4):500–18. doi: 10.1111/mpp.12770 (PMC6422694; doi:10.1111/mpp.12770)
Supplement: Supplementary file 13 — Table S1 Potential interacting protein list of MoPdeH identified by yeast two‐hybrid screen. [file MPP-20-500-s013.doc]

Table S1. Potential interacting protein list of MoPdeH identified by yeast two-hybrid screen

| Protein ID | Description | | | Number of screen | | | Interaction | | Knock out | | | | Phynotype | Report |  |
| --- | --- | --- | --- | --- | --- | --- | --- | --- | --- | --- | --- | --- | --- | --- | --- |
| MGG_03699 | Inosine-5'-monophosphate dehydrogenase | | 12 | | | Yes | | | | Yes | No | | | No | |
| MGG_13697 | Hypothetical protein | | | 10 | | | Yes | | Yes | | | No | | No | |
| MGG_04719 | Guanine nucleotide-binding protein subunit beta-like protein, MoMip11 | 9 | | | Yes | | | Yes | | | | Yes | | Yes | |
| MGG_04971 | Hypothetical protein | | | 9 | | | Yes | | Yes | | | No | | No | |
| MGG_03186 | 1,4-alpha-glucan-branching enzyme | | | 7 | | | Yes | | Yes | | | No | | No | |
| MGG_00883 | STE/STE11 protein kinase, MoMck1 | | | 7 | | | Yes | | Yes | | | Yes | | Yes |  |
| MGG_00703 | MAS3 protein | | | 7 | | | No | | - | | | - | | No |  |
| MGG_03663 | Hypothetical protein | | | 6 | | | No | | - | | | - | | No |  |
| MGG_00175 | 6-phosphogluconate dehydrogenase | | | 6 | | | Yes | | No | | | - | | No | |
| MGG_09830 | AAA family ATPase | | | 6 | | | Yes | | Yes | | | No | | No | |
| MGG_08895 | fructose-1,6-bisphosphatase | | | 6 | | | Yes | | Yes | | | No | | No | |
| MGG_06035 | FK506-binding protein 1B | | | 2 | | | No | | - | | | - | | No | |
| MGG_08904 | Hypothetical protein | | | 2 | | | No | | - | | | - | | No | |
| MGG_05338 | Hypothetical protein | | | 2 | | | No | | - | | | - | | No | |
| MGG_02061 | CAMK protein kinase | | | 2 | | | No | | - | | | - | | No | |
| MGG_15053 | Histone-lysine N-methyltransferase | | | 1 | | | No | | - | | | - | | No | |
| MGG_12467 | Hypothetical protein | | | 1 | | | No | | - | | | - | | No | |
| MGG_01712 | Isocitrate dehydrogenase subunit 2 | | | 1 | | | No | | - | | | - | | No | |
| MGG_16856 | Hypothetical protein | | | 1 | | | No | | - | | | - | | No | |
