# Supplementary material for: MoImd4 mediates crosstalk between MoPdeH‐cAMP signalling and purine metabolism to govern growth and pathogenicity in Magnaporthe oryzae
Source: Mol Plant Pathol. 2019 Jan 11;20(4):500–18. doi: 10.1111/mpp.12770 (PMC6422694; doi:10.1111/mpp.12770)
Supplement: Supplementary file 14 — Table S2 Appressorium formation on the hydrophobic surface of the wild‐type strain in the presence of mycophenolic acid (MPA). [file MPP-20-500-s014.doc]

Table S2. Appressorium formation analysis on the hydrophilic surface of the wild type under the treatments of different concentrations of MPA.

| Strain | 4 h | 8 h | 12 h | 24 h |
| --- | --- | --- | --- | --- |
| Guy11 | 39.0 ± 2.6 | 92.0 ± 0.1 | 90.3 ± 2.1 | 93.0 ± 2.6 |
| Guy11+ 1 μg/ml MPA | 34.0 ± 5.6 | 90.7 ± 4.2 | 93.7 ± 2.1 | 94.3 ± 0.6 |
| Guy11+ 5 μg/ml MPA | 37.3 ± 2.5 | 92.7 ± 2.1 | 94.7 ± 1.1 | 96.7 ± 0.1 |
| Guy11+ 10 μg/ml MPA | 37.3 ± 4.5 | 89.7 ± 3.5 | 93.7 ± 2.5 | 97.0 ± 0.1 |

Different concentrations of MPA (1 μg/ml, 5 μg/ml and 10 μg/ml) to analysis appressorium formation at 4 h, 8h, 12h and 24h. At least 100 spores were counted for each concentration.

±SD was calculated from three repeated experiments.
