# Supplementary material for: MoImd4 mediates crosstalk between MoPdeH‐cAMP signalling and purine metabolism to govern growth and pathogenicity in Magnaporthe oryzae
Source: Mol Plant Pathol. 2019 Jan 11;20(4):500–18. doi: 10.1111/mpp.12770 (PMC6422694; doi:10.1111/mpp.12770)
Supplement: Supplementary file 15 — Table S3 Primers used in this study. [file MPP-20-500-s015.docx]

**Table S3. Primers used in this study**

| **Primer name** | **Sequence (5’-3’)** | **Remark** |
| --- | --- | --- |
| *IMD4* KO-F1 | TAAAAGCTTGTGCCAATCGCAGTAACGATTA | amplify *MoIMD4* 5’ flank sequence |
| *IMD4* KO-F2 | TAAGAATTCCGTGAACTAAACATGACTTGTTA | amplify *MoIMD4* 3’ flank sequence |
| *IMD4* KO-F3 | TAAGCGGCCGCGTGGCAATACGAAAAAAAGATG | amplify *MoIMD4* 5’ flank sequence |
| *IMD4* KO-F4 | TAAGAGCT'CGTGAGCAGTAATGATGGTGGT | amplify *MoIMD4* 3’ flank sequence |
| *IMD4* BN-C1 | TTCCACCGTCATGATGGGTGG | Verification of *MoIMD4* deletion |
| *IMD4* BN-C2 | AGCGTAAAGCTTCTTCTCGTAA | Verification of *MoIMD4* deletion |
| *IMD4* BY | ATATTCGCTCTTAGCACGGCTG | Verification of *MoIMD4* deletion |
| *IMD4* HB-F | ACTCACTATAGGGCGAATTGGGTACTCAAATTGGTTATATTCGCTCTTAGCACGGCTG | *MoIMD4* complementation |
| *IMD4* HB -R | CACCACCCCGGTGAACAGCTCCTCGCCCTTGCTCACAGCGTAAAGCTTCTTCTCGTAA | *MoIMD4* complementation |
| 32a- *IMD4*-F | TAAGAATTCATGCCTGCAACAAACACCCAAG | Construction of *MoIMD4*−His |
| 32a- *IMD4*-R | TAAGCGGCCGCAGCGTAAAGCTTCTTCTCGTAA | Construction of *MoIMD4*−His |
| RT-*IMD4*-F | CTTTTCGGAGGGTGATAGCGT | qRT-PCR Primer of *MoIMD4* |
| RT-*IMD4*-R | AGCGTAAAGCTTCTTCTCGTAA | qRT-PCR Primer of *MoIMD4* |
| AD-*IMD4*-F | CCAGATTACGCTCATATGATGCCTGCAACAAACACCCAAG | Construction of pGADT7-*MoIMD4* |
| AD-*IMD4*-R | ATGCCCACCCGGGTGGAATTCTCAAGCGTAAAGCTTCTTCTCGT | Construction of pGADT7-*MoIMD4* |
| BD-*PDEH*-F | TAAGGATCCATGGAGAATGCTGCCTGCAAT | Construction of pGBKT7-*MoPDEH* |
| BD-*PDEH*-R | TAACTCGAGACCAGCAGTGTCGGCAGCCGA | Construction of pGBKT7-*MoPDEH* |
| pHZ126-*PDEH*-F | CTATAGGGCGAATTGGGTACTCAAATTGGTTATGGAGAATGCTGCCTGCAAT | Construction of *MoPDEH-*Flag |
| pHZ126-*PDEH*-R | CTTTATAATCACCGTCATGGTCTTTGTAGTCACCAGCAGTGTCGGCAGCCGA | Construction of *MoPDEH-*Flag |
| *IMD4*-CYFP-F | CGACTCACTATAGGGCGAATTGGGTACTCAAA TTGATGCCTGCAACAAACACCCAAG | Construction of *MoIMD4-*CYFP |
| *IMD4*-CYFP-R | GTTCGGGATCTTGCAGGCCGGGCGAGCGTAAAGCTTCTTCTCGTAA | Construction of *MoIMD4-*CYFP |
| *PDEH*-NYFP-F | CGACTCACTATAGGGCGAATTGGGTACTCAAATTGATGGAGAATGCTGCCTGCAAT | Construction of *MoPDEH-*NYFP |
| *PDEH*-NYFP-R | GCTCACCATCGTGGCGATGGAGCGACCAGCAGTGTCGGCAGCCGA | Construction of *MoPDEH-*NYFP |
| pYES2-*MoIMD4*-F | TAAGAATTCATGCCTGCAACAAACACCCAAG | Construction of pYES2-*MoIMD4* |
| pYES2-*MoIMD4*-R | TAAGCGGCCGCTCAAGCGTAAAGCTTCTTCTCGT | Construction of pYES2-*MoIMD4* |
| Rubq1 LL | GTGGTGGCCAGTAAGTCCTC | quantitative RT-PCR analysis |
| Rubq1 RR | GGACACAATGATTAGGGATCA | quantitative RT-PCR analysis |
| 28S rDNA LL | TACGAGAGGAACCGCTCATTCAGATAATTA | quantitative RT-PCR analysis |
| 28S rDNA RR | TCAGCAGATCGTAACGATAAAGCTACTC | quantitative RT-PCR analysis |
| T268AR269A- F2 | CCAATGGCAGCAGCGCACAGAA | Construction of T268AR269A-GFP and TR268269AA-His |
| T268AR269A - F3 | TTCTGTGCGCTGCTGCCATTGGCGCTGCACCAGAGGACAAGGTGCGCCTGCA | Construction of T T268AR269A -GFP and T268AR269A -His |
| D290A-F2 | CAGGATAACAACGTCCAGGCCAG | Construction of D290A-GFP and D290A-His |
| D290A-F3 | CTGGCCTGGACGTTGTTATCCTGGCCAGCAGCCAGGGCAACAGCATCTA | Construction of D290A-GFP and D290A-His |
| G340AG342A-F2 | AATACGAAGTCCATCAACACCAG | Construction of G340AG342A -GFP and G340AG342A -His |
| G340AG342A -F3 | CTGGTGTTGATGGACTTCGTATTGCAATGGCAAGCGGAAGTGCCTGCATTACCCA | Construction of GG340,342AA-GFP and GG340,342AA-His |
| S345AC347A-F2 | TCCGCTTCCCATGCCAATACGAA | Construction of S345AC347A -GFP and S345AC347A -His |
| S345AC347A -F3 | TTCGTATTGGCATGGGAAGCGGAGCTGCCGCCATTACCCAGGAGGTTATGGCCGT | Construction of S345AC347A -GFP and S345AC347A -His |
| D380AG382A -F2 | AGCGATGCAAGGAACACCGAACC | Construction of D380AG382A -GFP and D380AG382A -His |
| D380AG382A-F3 | GGTTCGGTGTTCCTTGCATCGCTGCCGGAGCTATTCAAAACGTGGGTCACATCGT | Construction of D380AG382A -GFP and D380AG382A -His |
| G403A-F2 | CATCATGACGGTGGAAGCACCAA | Construction of G403A-GFP and G403A-His |
| G403A-F3 | TTGGTGCTTCCACCGTCATGATGGCTGGTCTGCTAGCTGGTACTACCGA | Construction of G403A-GFP and G403A-His |
| Y428A-F2 | GGCCTTGACGAGCTTGCCTTCAC | Construction of Y428A-GFP and Y428A -His |
| Y428A-F2 | GTGAAGGCAAGCTCGTCAAGGCCGCCCGTGGCATGGGCAGCATCGACGC | Construction of Y428A-GFP and Y428A -His |
| M431AG432A-F2 | GCCACGGTAGGCCTTGACGAGCT | Construction of M431AG432A -GFP and M431AG432A -His |
| M431AG432A -F3 | AGCTCGTCAAGGCCTACCGTGGCGCGGCCAGCATCGACGCCATGCAAGACAA | Construction of M431AG432A -GFP and M431AG432A -His |
| R458AY459A-F2 | GGCAGTGCCAGCATTACTTTGCT | Construction of R458AY459A -GFP and R458AY459A -His |
| R458AY459A -F3 | AGCAAAGTAATGCTGGCACTGCCGCGGCCTTTTCGGAGGGTGATAGCGTCCT | Construction of R458AY459A -GFP and R458AY459A -His |
| Q470A-F2 | TGCAACCAGGACGCTATCACCCT | Construction of Q470A-GFP and Q470A -His |
| Q470A-F3 | AGGGTGATAGCGTCCTGGTTGCAGCGGGCGTTTCTGGCGCCGTGGCTCA | Construction of Q470A-GFP and Q470A -His |
| ΔCBS1-F2 | TAGAATGAAGCCGTTCTCGTAG | Construction of ΔCBS1-GFP and ΔCBS1-His |
| ΔCBS1-F3 | CTACGAGAACGGCTTCATTCTAGTCAACACCAAGGTCGCCGAT | Construction of ΔCBS1-GFP and ΔCBS1-His |
| ΔCBS2-F2 | AGTGACCATAACATCGGCGAC | Construction of ΔCBS2-GFP and ΔCBS2-His |
| ΔCBS2-F3 | GTCGCCGATGTTATGGTCACTTTCCCCCTGGCCTCCAAGCTC | Construction of ΔCBS2-GFP and ΔCBS2-His |
| ΔCBS1CBS2-F2 | GAGCTTGGAGGCCAGGGGGAATAGAATGAAGCCGTTCTCGTAG | Construction of ΔCBS1CBS2-GFP and ΔCBS1CBS2-His |
| ΔCBS1CBS2-F3 | CTACGAGAACGGCTTCATTCTATTCCCCCTGGCCTCCAAGCTC | Construction ofΔCBS1CBS2-GFP and ΔCBS1CBS2-His |
| 4T-2-*PDEH*-F1 | TAAGGATCCATGGAGAATGCTGCCTGCAATT | Construction of *MoPDEH*-GST |
| 4T-2-*PDEH*-F2 | TAAACTCGAGCGACCAGCAGTGTCGGCAGCCGAT | Construction of *MoPDEH*-GST |
